# BX795-Organic Acid Coevaporates: Evaluation of Solid-State Characteristics, In Vitro Cytocompatibility and In Vitro Activity against HSV-1 and HSV-2

**DOI:** 10.3390/pharmaceutics13111920

**Published:** 2021-11-12

**Authors:** Yogesh Sutar, Tejabhiram Yadavalli, Sagar Kumar Paul, Sudipta Mallick, Raghuram Koganti, Harsh Chauhan, Abhijit A. Date, Deepak Shukla

**Affiliations:** 1 Department of Pharmaceutical Sciences, The Daniel K. Inouye College of Pharmacy, University of Hawaii Hilo, Hilo, HI 96720, USA; ybsutar@hawaii.edu (Y.S.); smallick@hawaii.edu (S.M.); 2 Department of Ophthalmology and Visual Sciences, University of Illinois at Chicago, Chicago, IL 60612, USA; yteja@uic.edu (T.Y.); rkogan3@uic.edu (R.K.); 3 Department of Pharmaceutical Sciences, Creighton University School of Pharmacy and Health Profession, 2200 California Plaza, Omaha, NE 68710, USA; sagarkumarpaul@creighton.edu (S.K.P.); harshchauhan@creighton.edu (H.C.); 4 Department of Tropical Medicine, Medical Microbiology and Pharmacology, John A. Burns School of Medicine, University of Hawaii Manoa, Honolulu, HI 96813, USA; 5 Department of Microbiology and Immunology, University of Illinois at Chicago, Chicago, IL 60612, USA

**Keywords:** pK_a_, weakly basic, NMR, DSC, PXRD, herpes simplex virus, repurposing

## Abstract

BX795 is a TANK binding kinase-1 inhibitor that has shown excellent therapeutic activity in murine models of genital and ocular herpes infections on topical delivery. Currently, only the BX795 free base and its hydrochloride salt are available commercially. Here, we evaluate the ability of various organic acids suitable for vaginal and/or ocular delivery to form BX795 salts/cocrystals/co-amorphous systems with the aim of facilitating pharmaceutical development of BX795. We characterized BX795-organic acid coevaporates using powder X-ray diffractometry, Fourier-transform infrared spectroscopy (FT-IR), Raman spectroscopy, ^1^H-nuclear magnetic resonance spectroscopy, thermogravimetric analysis (TGA), and differential scanning calorimetry (DSC) to elucidate the interaction between BX795 and various organic acids such as taurine, maleic acid, fumaric acid, tartaric acid, and citric acid. Furthermore, using human corneal epithelial cells and HeLa cells, we evaluated BX795-organic acid coevaporates for in vitro cytocompatibility and in vitro antiviral activity against herpes simplex virus-type 1 (HSV-1) and type-2 (HSV-2). Our studies indicate that BX795 forms co-amorphous systems with tartaric acid and citric acid. Interestingly, the association of organic acids with BX795 improved its thermal stability. Our in vitro cytocompatibility and in vitro antiviral studies to screen suitable BX795-organic acid coevaporates for further development show that all BX795-organic acid systems, at a concentration equivalent to 10 µM BX795, retained antiviral activity against HSV-1 and HSV-2 but showed differential cytocompatibility. Further, dose-dependent in vitro cytocompatibility and antiviral activity studies on the BX795-fumaric acid system, BX795-tartaric acid co-amorphous system, and BX795-citric acid co-amorphous system show similar antiviral activity against HSV-1 and HSV-2 compared to BX795, whereas only the BX795-citric acid co-amorphous system showed higher in vitro cytocompatibility compared to BX795.

## 1. Introduction

Herpes simplex viruses (HSVs), also known as human herpesviruses, belong to the alpha-herpesviridae family and cause a variety of pathologies in humans. They are highly prevalent and ubiquitous in the developing world with seroprevalence reaching 100% in some cases [1,2,3]. While HSV type-1 (HSV-1) mainly causes mucocutaneous lesions in the orofacial region, it can also infect the eyes and lead to blindness in rare cases. On the other hand, HSV type-2 (HSV-2) predominantly causes painful warts and lesions in the anogenital region and can be sexually transmitted. Both HSV-1 and HSV-2 reside latently in the sensory ganglia of infected individuals and may reactivate from time to time causing a myriad of health problems. Infection with HSV in infants and adolescent humans can cause encephalitis which, if left untreated, can be lethal. HSV-1 and HSV-2 have been implicated in both short- and long-term neuronal loss which can lead to permanent brain damage and contribute to the onset of neurodegenerative diseases [1,2,3].

Currently, acyclovir (ACV) and its analogs such as ganciclovir, valaciclovir, and famciclovir are prescribed to subdue active viral replication. All of these drugs act via inhibiting the DNA replication cycle of the virus which leads to slower viral spread [1]. However, recent studies and multiple clinical reports have shown the emergence of drug-resistant strains that do not respond to ACV therapy. While there are other drugs such as cidofovir and foscarnet available to treat ACV-resistant HSV infections [1], there is a necessity to discover safer alternatives to ACV which target different aspects of the viral replication cycle.

Recently, we have shown that BX795, a TANK binding kinase-1 inhibitor, surprisingly limited HSV protein synthesis by inhibiting the hyper-phosphorylation of eukaryotic translation initiation factor 4E-binding protein 1 (4E-BP1) [4]. Our group has also shown that topically delivered BX795 can be repurposed for the prevention and treatment of HSV-1 and HSV-2 infection in murine models of ocular and genital herpes without any noticeable side effects [4,5,6,7]. However, to date, no reports exist on the characterization and pharmaceutical development of BX795 to facilitate its clinical translation for the treatment of genital and ocular herpes infections.

Salt/cocrystals/co-amorphous systems are widely explored strategies in the pharmaceutical industry to optimize physicochemical and/or biopharmaceutical properties of ionizable developmental compounds [8,9,10,11]. Appropriate selection of these strategies can improve/alter physicochemical properties such as melting point, crystallinity, physical and chemical stability, solubility, and biopharmaceutical properties such as dissolution rate, permeability, drug absorption, and bioavailability [8,9,10,11].

**Figure 1:**
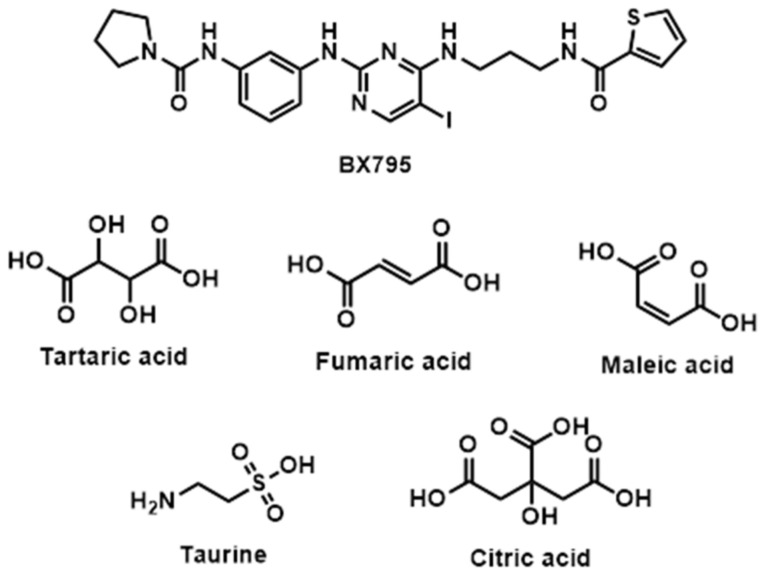
Chemical structure of BX795 and organic acids used for the preparation of co-amorphous systems.

BX795 is a weakly basic compound with an aminopyrimidine backbone which is available as a free base and BX795 hydrochloride salt. To date, no additional salt forms/cocrystals/co-amorphous systems of BX795 suitable for vaginal and ocular delivery have been developed and evaluated. Here, we evaluate the potential of various organic acids (Figure 1) suitable for vaginal and/or ocular delivery to yield BX795 salts/cocrystals/co-amorphous systems with improved pharmaceutical and biological characteristics. We report the solid-state, spectroscopic, and thermal characterization of BX795 and various BX795-organic acid coevaporates. Our studies show that tartaric acid and citric acid can form co-amorphous mixtures with BX795. Further, using thermogravimetric analysis (TGA), we show that certain BX795-organic acid co-amorphous systems have higher thermal stability compared to BX795. We also demonstrate that the organic acid used for the formation of BX795 co-amorphous system influences the in vitro cytocompatibility but retains the antiviral activity against HSV-1 and HSV-2. Finally, we show that the BX795-citric acid co-amorphous system has improved in vitro cytocompatibility without compromising the antiviral activity of BX795.

## 2. Materials and Methods

### 2.1. Materials

BX795 was purchased from Selleckchem.com (Houston, TX, USA). Fumaric acid, maleic acid, and L-(+)-tartaric acid were purchased from TCI Chemicals (Portland, OR, USA). Citric acid was purchased from VWR Life Science (Solon, OH, USA). Taurine was obtained from Alfa Aesar (Ward Hill, MA, USA). Hydrochloric acid (36.5–38%) and methanol were purchased from VWR Chemicals (Radnor, PA, USA), and all other chemicals used were of analytical grade unless otherwise indicated.

### 2.2. Viruses

HSV-1 and HSV-2 viruses mentioned in this study were a kind donation from Dr. Patricia Spear, Northwestern University, Chicago, IL, USA. HSV-1 strain 17-GFP is a mutant strain of HSV-1 with a cytomegalovirus (CMV) promoter to produce GFP protein upon entering the host cell nucleus. HSV-2 strain 333-GFP is a mutant strain of HSV-2 with a GFP gene on the CMV promoter to demonstrate successful infection of a host cell by the virus.

### 2.3. Cells

Human corneal epithelial cells (RCB1834 HCE-T) were obtained from K. Hayashi (National Eye Institute, Bethesda, MD, USA) and passaged in minimum essential medium (MEM Gibco/BRL, Carlsbad, CA, USA) supplemented with 10% fetal bovine serum (FBS-Sigma, St. Louis, MO, USA) and 1% penicillin/streptomycin (P/S) (Gibco, Amarillo, TX, USA). Human cervical epithelial (HeLa) cells were obtained from American Type Culture Collection and passaged in Dulbecco’s modified Eagle’s medium (DMEM) supplemented with 10% FBS and 1% P/S. African green monkey kidney (Vero) cells were obtained from Dr. Patricia Spear, Northwestern University and passaged in Dulbecco’s modified Eagle’s medium (DMEM) supplemented with 10% FBS and 1% P/S.

### 2.4. Synthesis of BX795-Organic Acid Coevaporates

#### 2.4.1. Synthesis of BX795-Tartrate/Maleate/Fumarate Coevaporates

A total of 50 mg (0.084 mmol) of BX795 and 0.084 mmol of tartaric acid/maleic acid/fumaric acid (1:1 molar) were dissolved in 10 mL of methanol and stirred for 2 h. The solvent was slowly evaporated on a rotary evaporator, and solid residue was kept for overnight drying in a vacuum oven.

*BX795-tartaric acid:* white solid; ^1^H NMR (400 MHz, DMSO-*d*_6_) δ 10.31 (s, 1H), 8.60 (t, *J* = 5.6 Hz, 1H), 8.22 (d, *J* = 12.9 Hz, 2H), 8.17 (s, 1H), 7.85 (s, 1H), 7.73 (d, *J* = 4.5 Hz, 2H), 7.18 (s, 3H), 7.12 (t, *J* = 4.4 Hz, 1H), 4.31 (s, 2H), 3.54 (dd, *J* = 5.3 Hz, 2H), 3.35 (s, 4H), 3.25 (q, *J* = c 6.3 Hz, 2H), 1.84–1.77 (m, 6H); ^13^C NMR (100 MHz, DMSO) δ 149.8, 112.9, 128.5, 131.0, 129.0, 116.1, 114.8, 128.3, 72.6, 40.2, 46.1, 37.1, 25.9, 29.0.

*BX795-maleic acid:* white solid; ^1^H NMR (400 MHz, DMSO-*d*_6_) δ 10.20 (s, 1H), 8.59 (t, *J* = 5.8 Hz, 1H), 8.21 (d, *J* = 13.4 Hz, 2H), 8.10 (s, 1H), 7.86 (s, 1H), 7.73 (d, *J* = 3.4 Hz, 2H), 7.17 (s, 3H), 7.12 (dd, *J* = 3.5, 4.8 Hz, 1H), 6.27 (s, 2H), 3.54 (q, *J* = 6.4 Hz, 2H), 3.35 (s, 4H), 3.26 (q, *J* = 6.8 Hz, 2H), 1.84–1.75 (m, 6H); ^13^C NMR (100 MHz, DMSO) δ 150.5, 112.8, 131.0, 128.4, 128.3, 129.0, 114.8, 116.0, 130.7, 40.2, 46.1, 37.1, 25.5, 29.0.

*BX795-fumaric acid:* white solid; ^1^H NMR (400 MHz, DMSO-*d*_6_) δ 10.24 (s, 1H), 8.59 (t, *J* = 5.6 Hz, 1H), 8.21 (d, *J* = 16.3 Hz, 2H), 8.12 (s, 1H), 7.86 (s, 1H), 7.73 (d, *J* = 4.1 Hz, 2H), 7.17 (s, 3H), 7.12 (t, *J* = 4.3 Hz, 1H), 6.63 (s, 2H), 3.54 (dd, *J* = 6.7 Hz, 2H), 3.35 (s, 4H), 3.26 (q, *J* = 6.2 Hz, 2H), 1.84–1.78 (m, 6H); ^13^C NMR (100 MHz, DMSO) δ 150.1, 112.8, 131.0, 128.4, 129.0, 114.8, 116.0, 128.3, 134.4, 40.2, 46.1, 37.1, 25.4, 29.0.

#### 2.4.2. Synthesis of BX795-Taurine Coevaporate

A total of 50 mg (0.084 mmol) of BX795 and 0.168 mmol of taurine (1:2 molar) were dissolved in 10 mL of methanol/water (7:3) and stirred for 2 h. The solvent was slowly evaporated on a rotary evaporator, and solid residue was kept for overnight drying in a vacuum oven.

*BX795-taurine:* white solid; ^1^H NMR (400 MHz, DMSO-*d*_6_) δ 10.19 (s, 1H), 8.58 (t, *J* = 5.9 Hz, 1H), 8.20 (d, *J* = 14.2 Hz, 2H), 8.10 (s, 1H), 7.86 (s, 1H), 7.74 (m, 6H), 7.18 (s, 3H), 7.13 (t, *J* = 4.5, 1H), 3.52 (q, *J* = 6.5 Hz, 2H), 3.36 (s, 4H), 3.27 (q, *J* = 6.5 Hz, 2H), 3.06 (m, 4H), 2.73 (t, *J* = 6.4 Hz, 4H), 1.86–1.75 (m, 6H). ^13^C NMR (100 MHz, DMSO) δ 150.5, 112.9, 131.0, 128.5, 114.8, 116.0, 40.2, 46.1, 37.1, 36.5, 48.0, 25.5, 29.0.

#### 2.4.3. Synthesis of BX795 Dihydrochloride Salt

A total of 50 mg (0.084 mmol) of BX795 and 0.168 mmol of 1M HCl solution (1:2 molar) were dissolved in 10 mL of methanol and stirred for 2 h. The solvent was slowly evaporated on a rotary evaporator, and solid residue was kept for overnight drying in a vacuum oven.

*BX795 dihydrochloride:* white solid; ^1^H NMR (400 MHz, DMSO-*d*_6_) δ 10.54 (brs, 1H), 8.65 (t, *J* = 5.8 Hz, 1H), 8.36 (t, *J* = 6.1 Hz, 1H), 8.27 (d, *J* = 15.7 Hz, 2H), 7.86 (s, 1H), 7.75 (dd, *J* = 3.6, 6.1 Hz, 2H), 7.20 (m, 3H), 7.13 (t, *J* = 4.4 Hz, 1H), 3.56 (q, *J* = 6.7 Hz, 2H), 3.36 (s, 4H), 3.26 (q, *J* = 6.5 Hz, 2H), 1.85–1.77 (m, 6H). ^13^C NMR (100 MHz, DMSO) δ 148.4, 112.9, 128.5, 131.0, 116.3, 129.1, 114.8, 128.3, 40.4, 46.1, 37.1, 25.4, 28.9.

#### 2.4.4. Synthesis of BX795-Citric Acid Coevaporate

A total of 50 mg (0.126 mmol) of BX795 and 0.084 mmol of citric acid (1.5:1 molar) were dissolved in 10 mL of methanol and stirred for 2 h. The solvent was slowly evaporated on a rotary evaporator, and solid residue was kept for overnight drying in a vacuum oven.

*BX795-citric acid:* white solid; ^1^H NMR (400 MHz, DMSO-d_6_) δ 10.22 (s, 1H), 8.58 (t, J = 5.6 Hz, 1H), 8.21 (d, J = 12.5 Hz, 2H), 8.14 (brs, 1H), 7.86 (s, 1H), 7.73 (d, J = 5.2, 3H), 7.17 (s, 3H), 7.12–7.08 (m, 1H), 3.53 (d, J = 6.3, 4H), 3.35 (s, 5H), 3.27 (q, J = 6.5, 4H), 2.76 (d, J = 15.5, 2H), 2.65 (d, J = 15.4 Hz, 2H), 1.84–1.76 (m, 9H). ^13^C NMR (101 MHz, DMSO-d_6_) δ 149.8, 112.9, 131.0, 128.4, 129.1, 116.1, 114.9, 128.3, 40.2, 46.2, 37.1, 43.2, 25.4, 29.0.

### 2.5. Fourier Transform-Infrared (FTIR) Spectroscopy

FTIR spectroscopy measurements were performed using a Fourier transform infrared (FTIR) spectrophotometer (Thermo Scientific, Waltham, MA, USA) equipped with a diamond attenuated total reflection (ATR) unit. FTIR spectra were obtained in transmission mode from 4000 to 500 cm^−1^ and with an average of 32 scans for background and each sample.

### 2.6. Raman Spectroscopy

All the samples were analyzed for any vibrational change by Raman spectroscopy (Bruker-Senterra Dispersive Raman Microspectrometer, Bruker Corp., Billerica, MA, USA). A small amount of sample was mounted on a glass slide and placed on the stage of the Raman micro-spectrometer. The lens was focused by a 20× objective with an aperture of 50 × 1000 μm on the sample. This sample was then subjected to a laser beam with a wavelength of 785 nm and a laser power of 50 mW. A resolution of 9–15 cm^−1^ was used for a spectral range of 50 to 3500 cm^−1^. The data were acquired with 5 s of integration time and 2 co-additions.

### 2.7. Nuclear Magnetic Resonance (NMR) Spectroscopy

Nuclear magnetic resonance (NMR) spectra were obtained using a Bruker Avance Digital 400 MHz NMR spectrometer (Bruker Corp., Billerica, MA, USA) coupled to a BACS 1 automatic sample changer. The spectrometer is equipped with a 5-mm PABBO BB-1H/D Z-GRD probe. The 1H spectrum of the purified products (6–8 mg) was recorded in dimethyl sulfoxide-*d_6_* (ACROS ORGANICS, 99.5% D) with an average of 16 scans for each sample. Chemical shifts were reported in ppm with residual undeuterated solvent peaks as the internal reference for ^1^H NMR: DMSO (2.50 ppm).

### 2.8. Powder X-ray Diffraction (PXRD) Studies

All the samples were analyzed for crystallinity utilizing using Rigaku Smartlab (Rigaku Corp., Tokyo Japan) and PANalytical Empyrean diffractometers (Malvern PANalytical Inc., Westborough, MA, USA), both using Cu K_α radiation (Wavelength = 1.541 Å). The data were collected using a Bragg-Brentano focusing geometry instrument with identical incident and receiving slits and employing unique 1D silicon strip detectors, D/teX Ultra 250 and PIXcel 3D, respectively. In both diffractometers, the sample, spread on a flat holder, remained stationary while both source and detector moved continuously in a coupled way. The data were collected within the range 5–60° 2θ, continuously scanning θ/2θ scan at 5 deg/min with steps of 0.010°. PXRD analyses on samples were also performed after 1 month of storage at room temperature.

### 2.9. Thermogravimetric Analysis (TGA)

All the samples were analyzed by a TGA-50 Shimadzu thermogravimetric analyzer (Shimadzu America Inc., Columbia, MD, USA) for thermal degradation. A sample of 3–5 mg was accurately weighed and taken into an aluminum pan and loaded onto the sample holder. Nitrogen was purged at a rate of 20 mL/min, and samples were heated from room temperature to 300 °C at a rate of 10 °C/min.

### 2.10. Differential Scanning Calorimetry (DSC)

Thermograms were recorded for pure compounds and the prepared salts by utilizing Q2000 Modulated DSC (TA Instruments, New Castle, DE, USA) equipped with a refrigerated cooling system (RCS90). The instrument was calibrated with indium. Samples of 3–10 mg were taken in a Tzero aluminum pan and hermetically crimped for the run. An empty aluminum pan was used as a reference and nitrogen was purged at a rate of 20 mL/min to keep the atmosphere inert. TA Universal analysis software (Universal Analysis 2000, TA instruments, New Castle, DE, USA) was used to analyze the thermograms. A simple 5 °C/min heating cycle from 30 to 200 °C was used to observe the thermal events.

### 2.11. MTT Assay

An MTT (3-(4,5-dimethylthiazol-2-yl)-2,5-diphenyltetrazolium bromide) viability assay on HCE and HeLa cell lines using various concentrations of BX795 salts was performed after 24 h of incubation. Briefly, cells were plated at a density of 1 × 104 per well in a 96-well plate overnight. The following morning, concentrations starting at 50 µM were two-fold serially diluted and added to cell monolayers in whole media for 24 h. At the end of incubation, MTT (0.5 mg/mL in whole media) was added to cells and incubated for 3 h to allow formazan crystal formation. Acidified isopropanol (0.1% glacial acetic acid *v*/*v*) was added to cells to dissolve the formazan crystals. Dissolved violet crystals were transferred to a new 96-well plate and analyzed by a microplate reader (Bio Tek Instruments, Winooski, VT, USA) at 550 nm.

### 2.12. Western Blotting

Immunoblotting analysis was performed using the methods mentioned previously [12]. Samples were blotted for the presence of HSV-1/HSV-2 gB using 1:1000 diluted gB antibody (Abcam-6506, Cambridge, MA, USA) in 5% skim milk. GAPDH (Proteintech 10494-1-AP, Chicago, IL, USA) was used as a loading control.

### 2.13. Flow Cytometry

Cell collection, cytometry, and analysis were performed using previously reported methods [12]. All the data collected from the instrument were analyzed in FlowJo v.10 (Ashland, OR, USA).

### 2.14. Plaque Assay

Infected cells were collected and lysed using a probe sonication instrument for 15 s on ice. This process releases the viruses by lysing the plasma membrane of the infected cells. These infectious samples were then serially diluted 10-fold in OptiMEM (Gibco, Fisher Scientific, Hampton, NH, USA) before overlaying them on a confluent monolayer of Vero Cells to perform a titration experiment. At 2 hpi, the overlaid media was aspirated, and 5% methylcellulose laden DMEM was added. At 72 hpi, the cells were fixed using 100% methanol and stained with crystal violet to visualize plaques. Plaques were counted for individual dilutions and recorded for analysis.

### 2.15. Imaging

Cells were imaged using a Lionheart LX instrument. This imaging system was used to image GFP-positive cells to visualize the extent of infection in treated samples. This imaging system also comes with Gen5 software (Charlotte, NC, USA) that enables cellular statistics to count the total number of cells in addition to fluorescent protein-tagged cells. The software automatically detects the baseline fluorescence level to determine true positive cells within a given population. These datasets were individually recorded for each experiment to determine EC_50_ values.

### 2.16. Statistics

GraphPad Prism version 4.0 (GraphPad Software, San Diego, CA, USA) was used for statistical analysis of each group. *p* < 0.05 was considered as a significant difference among mock-treated and treated groups.

## 3. Results

### 3.1. Powder X-ray Diffraction (PXRD) Showed the Formation of BX795-Organic Acid Salt/Cocrystals/Co-Amorphous Systems

**Figure 2:**
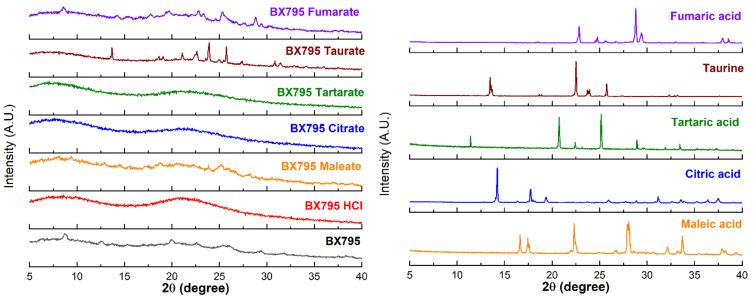
X-ray powder diffraction (XRPD) characterization of BX795 (processed using solvent evaporation method similar to BX795-organic acid coevaporates) and BX795-organic acid coevaporates. XRPD of BX795-citrate and BX795-tartrate indicate the formation of co-amorphous systems. BX795 HCl shows amorphous nature. The other BX795-organic acid coevaporates show crystalline or partially crystalline nature.

The pure BX795 and its organic acid coevaporates were characterized by PXRD (Figure 2) to check their solid-state characteristics. The unique diffraction patterns were used to evaluate the formation of salts/cocrystals/co-amorphous systems. Commercially available BX795 appeared to be partially crystalline, and it showed diffraction peaks at 2θ of 8.8°, 18.9°, 22.6°, 26.0°, and 29.3°. All the pure organic acids showed sharp diffraction peaks indicating their crystalline form. BX795 HCl showed amorphous nature. BX795-fumaric acid coevaporate, BX795-maleic acid coevaporate, and BX795-taurine coevaporate showed diffraction peaks confirming their crystalline or partially crystalline nature. These sharp diffraction peaks corresponded to either pure drug or organic acid. Some diffraction peaks from both BX795 and their respective organic acids were missing indicating a partial change in crystal arrangement. This observation confirms the formation of BX795 salts/cocrystals with their unique crystal lattice and modified solid-state properties. BX795-fumaric acid coevaporate showed diffraction peaks corresponding to BX795 as well as fumaric acid. Finally, BX795-tartaric acid coevaporate and BX795-citric acid coevaporate were found to be amorphous, as they showed a halo diffraction pattern instead of sharp diffraction peaks indicating the formation of co-amorphous systems. 

### 3.2. Spectroscopic Studies Showed Significant Interaction between Organic Acids and BX795

Pure BX795, organic acids, and BX795-organic acid coevaporates were characterized using various spectroscopic techniques such as Fourier-transform infrared (FTIR) spectroscopy, Raman spectroscopy, and nuclear magnetic resonance (NMR) spectroscopy.

**Figure 3:**
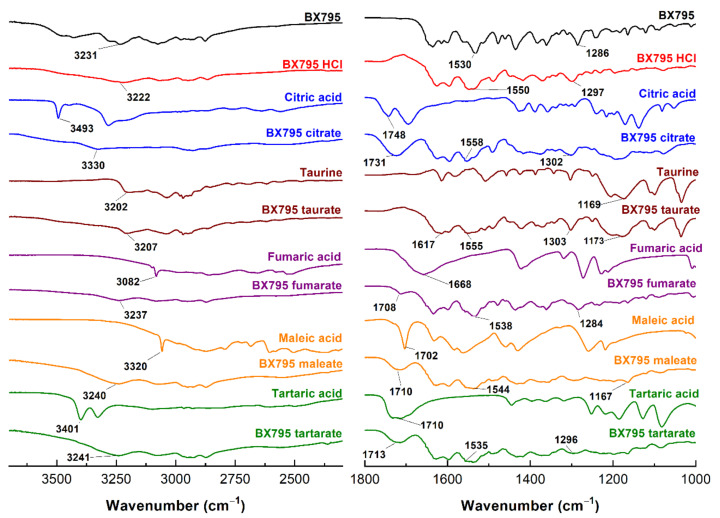
FT-IR spectra of BX795, pure organic acids, and BX795-organic acid systems. The FT-IR spectra of BX795-organic acid systems showed shifts in the characteristic BX795 bands corresponding to the –NH stretching vibration (3231 cm^−1^), –NH bending (1530 cm^−1^), and C-N stretching (1286 cm^−1^) of a secondary or tertiary amine.

The FTIR spectra of BX795-organic acids (or hydrochloric acid) indicated a significant interaction between BX795 and acidic counterparts. The broad hump-like band from 2500 to 3300 cm^−1^ in BX795-maleic acid/tartaric acid/fumaric acid coevaporate appeared due to –OH stretching of carboxylic acid (Figure 3). The band at 3231, 1530, and 1286 cm^−1^ in BX795 spectrum corresponds to the –NH stretching vibration, –NH bending, and C-N stretching of the secondary or tertiary amine, respectively, all of which shifted in the FTIR spectra of all BX795-organic acid coevaporates. The stretching vibration of the C=O (carboxylic acid) at around ~1700 cm^−1^ present in BX795-maleic acid/tartaric acid/citric acid/fumaric acid coevaporate showed the alterations compared to the spectra of corresponding acids (Figure 3 and Figure S1, Table 1).

The shifting in the values and decrease in the intensity implies that the C=O participated in strong hydrogen bond formation. However, in the case of taurine, a strong band at 1169 cm^−1^ attributed to the S=O stretching of sulfonic acid showed shifting and decreased intensity in its BX795 salt (1173 cm^−1^), confirming its involvement in hydrogen bond formation (Figure 3, Table 1).

The Raman spectra of BX795, organic acid and their corresponding BX795 coevaporates are shown in Figure S2. The Raman spectrum of BX795 has a peak at 1084 cm^−1^ attributed to C–N of the tertiary amine, whereas, in its corresponding BX795 coevaporates, the peak shifted to 1090 cm^−1^. The Raman spectra of organic acids (tartaric acid, maleic acid, fumaric acid, and citric acid) showed C=O stretching at ~1690 cm^−1^. This peak shifted or merged in the spectrum (~1633 cm^−1^ for C=O of amide) of their corresponding BX795 coevaporates. The shift or disappearance of the C–N stretching and C=O stretching mode in the BX795-organic acid coevaporate spectra indicate the strong interaction (hydrogen bond formation) between the carboxylic group of the organic acid and tertiary amine of BX795. The Raman spectrum of taurine showed a characteristic peak of S=O stretching at 1032 cm^−1^ which diminished and merged in BX795-taurine salt indicating the interaction between BX795 and taurine. Furthermore, a slight shift in amide N–H + C–H deformation (1285 cm^−1^ in BX795 Raman spectrum) indicated the intermolecular interaction between BX795 and corresponding acids. Thus, the Raman data supported the results from the FTIR spectroscopy.

We also characterized BX795-organic acid coevaporates using ^1^H & HSQC (2D) NMR. The ^1^H NMR of BX795, organic acids, and BX795-organic acid coevaporates confirmed the molecular interaction between BX795 and organic acids (Figures S3–S16). The shifts in the NMR signals of characteristic protons (Table 2) further support the interaction between BX795 and organic acids. The signal for the carboxyl proton present in organic acids disappeared in their corresponding coevaporates. The chemical shift was observed in the secondary amine (–NH *δ*_H_: 8.59 ppm for BX795) of BX795-citric acid/tartaric acid/hydrochloride (Table 2), whereas disappearance or decrease in the intensity of pyrimidine –CH proton and carbon signal was observed in all BX795 coevaporates (Figures S4, S6, S8, S10, S12, S14 and S16 and Table 2). The 2D spectrum of BX795 and its corresponding organic acid coevaporates revealed carbon-proton correlations (Figures S3, S5, S7, S9, S11, S13 and S15). The disappearance of signal from neighboring proton (–CH) of the tertiary amine of pyrimidine (BX795: *δ*_H_/*δ*_C_: 8.22/149.8) in BX795-organic acid coevaporates confirmed the protonation of the tertiary amine (Figures S3, S5, S7, S9, S11, S13 and S15). This may be due to the presence of bulky groups around pyrimidine –CH causing hindrance in the detection of –CH proton in the 2D NMR. However, in the case of HCl salt, because of less hindrance, –CH proton of pyrimidine signal appeared. Interestingly, in 2D/^1^H NMR of Bx795 taurate and Bx795.HCl, it was observed that there is a considerable move in –CH proton signal in pyrrolidine as well (BX795: 3.54 ppm, BX795-taurine: 3.52 ppm, BX795.HCl: 3.56 ppm) (Figures S12–S15). This may be because of 1:2 compositions of Bx795 and acid, which force it to protonate at both the tertiary amine sites. The 2D NMR studies confirmed the interaction between the carboxylic/sulfonate group of acids and tertiary amine of BX795. These interactions might be more pronounced in the case of the BX795-citric acid co-amorphous system because of the extra hydrogen acceptor group.

### 3.3. Thermal Characterization Studies on BX795-Organic Acid Coevaporates Showed Differential Solid-State Properties

**Figure 4:**
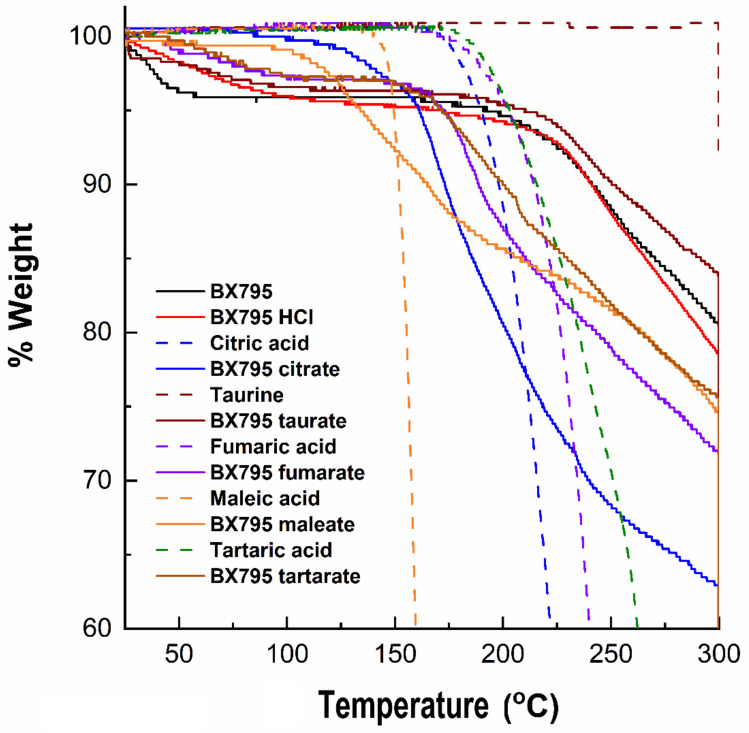
Thermogravimetric analysis of BX795 (commercially available), pure organic acids, and BX795-organic acid systems. BX795 showed ~4% loss in the mass at ~60 °C, whereas BX795-organic acid systems showed variable but improved thermal stability. Among BX795-organic acid systems, the temperature corresponding to 2% mass loss was the highest (~137 °C) for BX795-citrate indicating its thermal stability.

We evaluated BX795 and its coevaporates with organic acids using TGA to determine the thermal stability of various samples. TGA monitors the weight loss of samples as a function of an increase in the temperature and provides insight into the presence of solvent/moisture or drug degradation. The % degradation of BX795 and BX795-organic acid systems and the corresponding temperature are shown in Table 3. Commercially available BX795 showed a weight loss (~2%) which started from ~37 °C (Table 3), continued until 58 °C (~4% loss), and finally started degrading from ~200 °C (Figure 4). The initial weight loss could be a characteristic feature of the free base. Pure organic acid showed no moisture/solvent, and its degradation patterns were similar to those reported in the literature. The weight loss (3–4%) around 100 °C was also observed for the BX795 HCl, BX795 tartrate, and BX795 fumarate (Figure 4). Similar to BX795, all these salts degraded after 200 °C. However, BX795 maleate and BX795 citrate started degrading at ~100° and ~140°, respectively. This might be due to the degradation of pure malic acid (degradation starts at ~145°) and pure citric acid (degradation starts at ~180°). Overall, prepared BX795-organic acid coevaporates were stable and might have an advantage of greater thermal stability compared to pure BX795.

**Figure 5:**
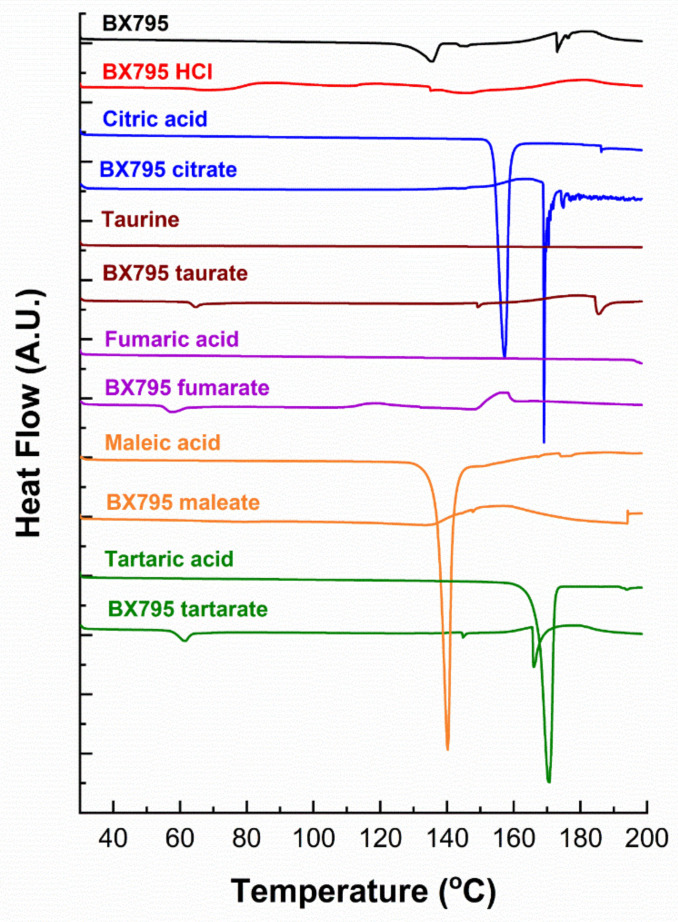
The differential scanning calorimetry (DSC) thermograms of BX795, pure organic acids, and BX795-organic acid coevaporates.

The differential scanning calorimetry (DSC) thermograms of BX795 and BX795-organic acid coevaporates are shown in Figure 5. BX795 showed a melting endotherm ~135.5°, confirming its crystalline nature (Figure 5). After the melting endotherm, the baseline shifts up and down indicating either the degradation of BX795 or a combination of uncharacterizable complex exothermic and endothermic events. Pure organic salts showed a sharp melting endotherm, similar to that reported in the literature. For crystalline salt, none of them showed a melting point similar to BX795, confirming the formation of salts with different thermal properties. BX795 HCl, fumarate, and maleate salts thermograms were inconclusive as only broad complex endothermic and exothermic transitions or a slight change in baseline was observed. For BX795 citrate salt, a sharp endotherm at 169.9℃ was observed. Other salts, such as BX795 tartrate and BX795 taurate showed a set of three endotherms around 60°, 150°, 180°. A similar endotherm near 60° was also observed for BX795 fumarate. These similarities in endotherms suggest some commonality in these prepared salts. Overall, all the prepared salts showed different thermal behavior compared to pure BX795 and further thermal characterization is required to assign and understand the specific endothermic and exothermic transitions associated with the salt forms.

### 3.4. BX795-Organic Acid Coevaporates Show Antiviral Activity at Concentration, Similar to BX795

**Figure 6:**
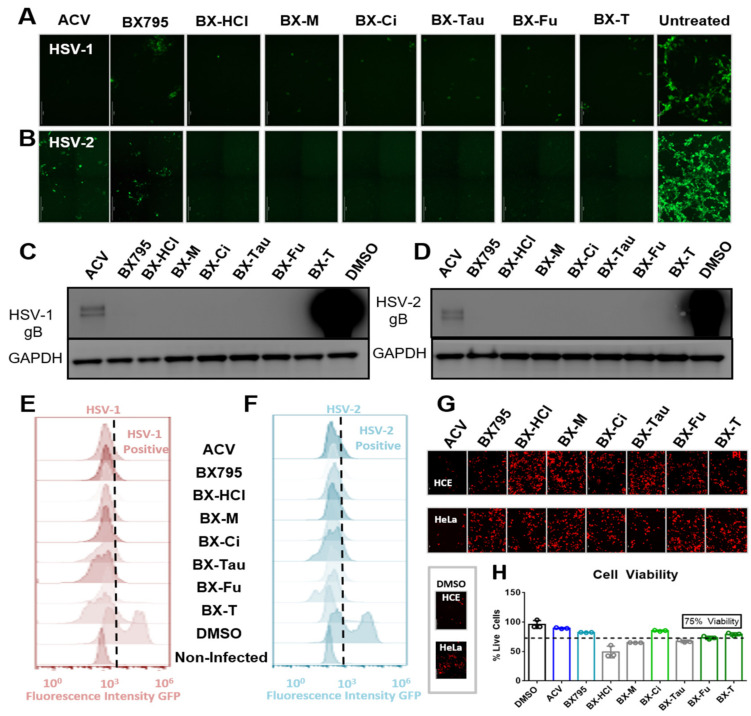
Antiviral activity and cellular toxicity of different BX795 salts: (**A**,**B**) Human corneal epithelial (HCE) cells and human cervical epithelial (HeLa) cells were infected with HSV-1 strain K26-GFP or HSV-2 strain 333-GFP, respectively, at a multiplicity of infection (MOI) of 0.1 for 2 h before treating them with 10 µM respective drug solutions. At 24 h post-infection, cells were imaged at 10X magnification for the presence of infected cells by detecting GFP-fluorescence-positive cells. (**C**,**D**) Infected cells treated with various drugs were collected at 24 h post-infection and processed for Western blotting analysis. HSV-1 or HSV-2 glycoprotein-B was immunoblotted to determine the extent of viral protein synthesis during the infection period. GAPDH was used as a loading control. (**E**,**F**) HCE and HeLa cells infected with GFP-reporter HSV-1 or HSV-2, respectively, were treated with shown drugs at 10 µM for 24 h, at which point cells were collected, fixed with 4% paraformaldehyde, and analyzed using flow cytometry. GFP-positive (infected) cells were spotted using non-infected cells as control fluorescence in the histograms. (**G**) HCE or HeLa cells were treated with shown drugs at a concentration of 10 µM for 24 h without viral infection. All cells were dyed with 10 µL/mL of 1.0 mg/mL propidium iodide (PI) solution to visualize dead cells. (**H**) An MTT assay was performed on these cells to detect cell viability compared to DMSO controls. All drugs that showed greater than 75% cell viability at 10 µM concentration were chosen for further study. (BX-HCl: BX795-dihydrochloride; BX-M: BX795-maleic acid; BX-Ci: BX795-citric acid; BX-Tau: BX795-taurate; BX-F: BX795-fumaric acid; BX-T: BX795-tartaric acid).

We tested the antiviral activity of all the salts previously mentioned on HSV-1 or HSV-2 infected human corneal epithelial (HCE) cells and human cervical epithelial (HeLa) cells, respectively. These studies were all performed at 10 µM concentration. ACV at 10 µM was used as positive control and an equivalent volume of DMSO was used as a negative control. Green fluorescence protein (GFP) producing reporter strains of HSV-1 and HSV-2 were used to evaluate the extent of infection. Our results showed that all the BX795-organic acid salt/cocrystals/co-amorphous systems, when used at a concentration equivalent to 10 µM BX795, were effective in inhibiting viral infection as seen by the absence of GFP in fluorescent images taken 24 h post-infection (hpi) (Figure 6A,B). Cell lysates of these samples immunoblotted for the presence of a late viral protein glycoprotein-B (gB) also showed no HSV-1 gB or HSV-2 gB (Figure 6C,D). Quantitative analysis of cells via flow cytometry for the presence of GFP-positive cells (infected) showed results similar to ACV treated cells (Figure 6E,F). HCE and HeLa cells were treated with a 10 µM concentration of ACV, BX795, and BX795-organic acid coevaporates for 24 h followed by the addition of propidium iodide (PI) to stain for dead cells (Figure 6G). These results showed that except for BX795-citric acid/tartaric acid/fumaric acid systems, the rest of the BX795-organic acid systems were slightly more toxic than BX795 alone. To quantitatively test the viability of cells at 10 µM concentration, we performed an MTT (3-(4,5-dimethylthiazol-2-yl)-2,5-diphenyltetrazolium bromide) assay. Our results showed that BX795 and BX795-citric acid/tartaric acid/fumaric acid systems had greater than or equal to 75% cell viability in HCE cells. Hence, these three BX795-organic acid systems were chosen for further analysis.

### 3.5. BX795-Citric Acid/Tartaric Acid/Fumaric Acid Coevaporates Are Tolerable and Provide Robust Antiviral Activity

**Figure 7:**
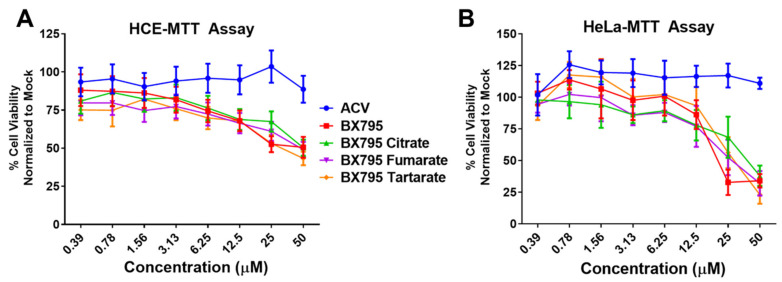

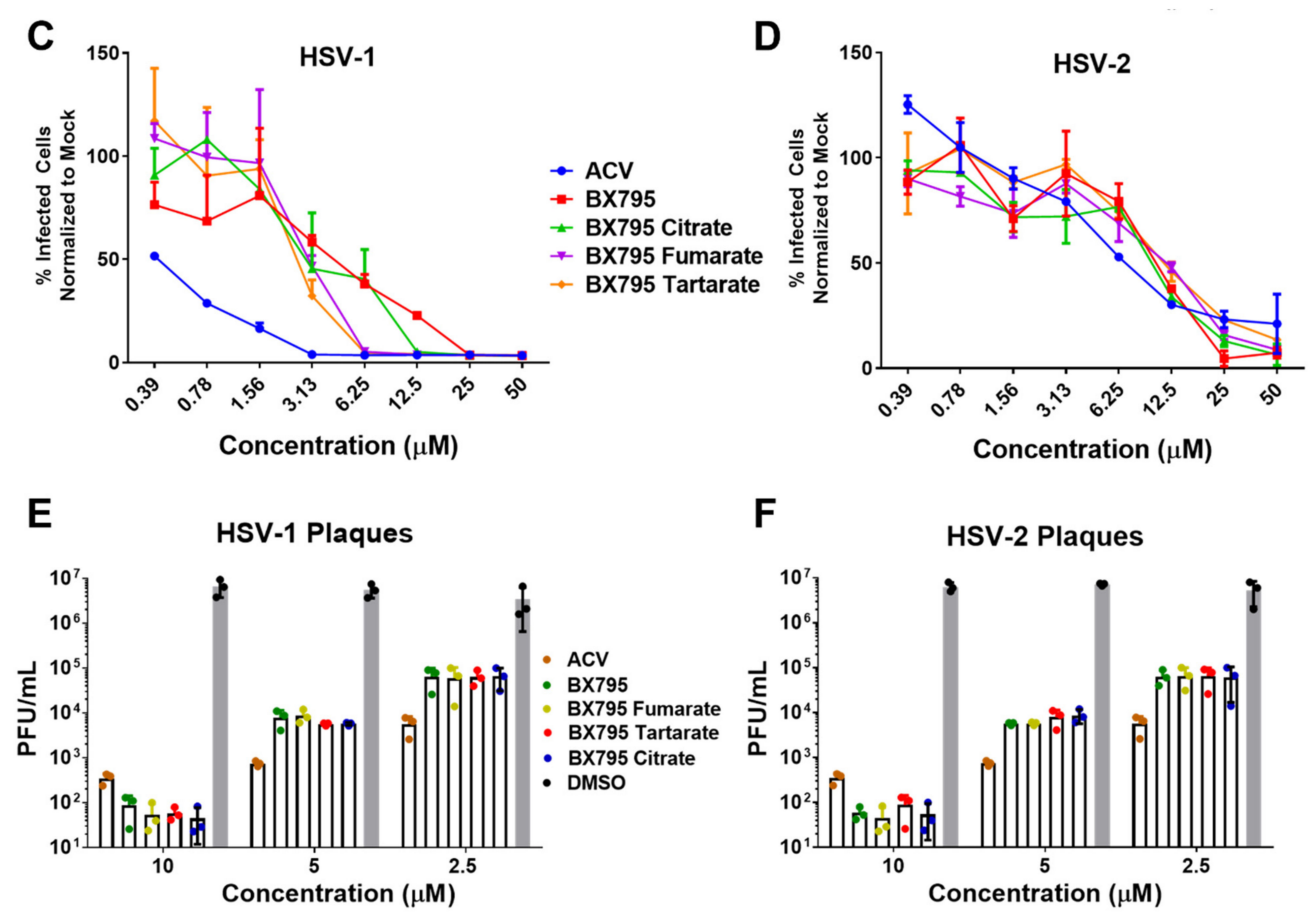
Cell viability and antiviral activity and of BX795-citrate, BX795-fumarate, and BX795-tartrate co-amorphous systems: (**A**,**B**) Human corneal epithelial (HCE) cells and human cervical epithelial (HeLa) cells were treated with various concentrations of BX795 salts using BX795, ACV, and DMSO as controls. At 24 h post-addition of drugs, cells were processed for the MTT assay as per manufacturer’s protocol, and the colorimetric readings were analyzed at 550 nm. (**C**,**D**) HCE and HeLa cells were infected with HSV-1 strain 17-GFP or HSV-2 strain 333-GFP, respectively, at a multiplicity of infection (MOI) of 0.1 for 2 h before treating them with specified drug concentrations. At 24 h post-infection, cells were imaged for the presence of infected cells by detecting GFP-fluorescence-positive cells using the Lionheart LX imaging system. The percentage of infected cells was calculated by comparing GFP-positive (infected) cells to total cells present in the imaged area, and line graphs were drawn accordingly. (**E**,**F**) HCE and HeLa cells infected with HSV-1 or HSV-2, respectively, were treated with ACV, BX795, BX795-organic acid coevaporates, or DMSO at 10, 5, or 2.5 µM concentration for 24 h, at which point cells were collected, lysed, and serially diluted and overlaid on Vero cells to perform a plaque assay. Plaques were counted 72 h after initiating the plaque assay by fixing cells with 100% methanol and dyeing with crystal violet.

After performing initial studies on the BX795-organic acid coevaporates at 10 µM concentration, we opted to carry out a more in-depth analysis of cellular toxicity and antiviral activity at different concentrations. To rule out contribution of organic acids used for the preparation of BX795-organic acid coevaporates towards cytotoxicity and/or antiviral activity, we first evaluated all organic acids for their cytocompatibility and antiviral activity. As anticipated, all organic acids were very well tolerated by HCE cells at least up to 100 µM and did not show any antiviral activity (Figure S17). For BX795-organic acid coevaporates, all the experiments were performed starting at 50 µM and serially diluted 2-fold until 0.3 µM concentration. MTT assays of the various BX795-organic acid coevaporates showed that the toxic concentration 50% (CC_50_) in HCE cells was above 50 µM concentration (Figure 7A). On the other hand, BX795, BX795-tartaric acid co-amorphous system, and BX795-fumaric acid system reached CC_50_ by 25 µM concentration in HeLa cells. However, BX795 citrate reached CC_50_ only at 50 µM concentration (Figure 7B). The inhibitory concentration 50% (EC_50_) was evaluated by counting infected (GFP-positive) cells within the total cell population. Our results show that all BX795-citric acid/tartaric acid/fumaric acid systems reached EC_50_ for HSV-1 in HCE cells and HSV-2 in HeLa cells at a concentration equivalent to 6.25 µM BX795 (Figure 7C,D). It should be noted that the GFP produced in these cells is an indicator of the number of cells infected and does not demonstrate the total number of infectious viral particles present in the treated cell. To evaluate the total number of infectious particles, we performed plaque assays using BX795 salts at 10, 5, and 2.5 µM concentrations. HCE or HeLa cells infected with HSV-1 or HSV-2, respectively, were treated with BX795-citric acid/tartaric acid/fumaric acid systems starting at 2 hpi. At 24 hpi, cells were collected, lysed, and overlaid on African green monkey kidney (Vero) cells at multiple Log10 fold dilutions to perform a plaque assay. Plaques counted from individual assays showed that even at 2.5 µM concentration, BX795-citric acid/tartaric acid/fumaric acid systems resulted in >2-log_10_ fold reduction in infectious viral particles (Figure 7E,F).

## 4. Discussion

Our previous preclinical studies on DMSO solubilized and locally delivered BX795 have shown strong evidence of efficacy in a murine model of genital and ocular herpes simplex virus infection [4,5,6,7]. While cosolvent-based (DMSO) solubilization was sufficient for initial evidence of efficacy, the pharmaceutical development of BX795 is highly warranted to facilitate its translation as a modality for topical treatment of genital and ocular herpes. Salt formation is a first-line pharmaceutical development strategy to improve the physicochemical and/or biopharmaceutical properties of ionizable drugs such as BX795. Currently, BX795 hydrochloride salt is commercially available but no pharmaceutical characterization data are available for this salt. To develop a salt form of a weakly basic compound such as BX795, a long list of FDA-approved counterions is available [8]. However, as BX795 is intended to be developed for topical vaginal and ocular delivery, the option of counterions to develop the salt form narrowed significantly. Organic carboxylic acids such as maleic acid, fumaric acid, tartaric acid, and citric acid have been previously used either for the development of salts of drugs intended for vaginal and/or ocular delivery or as a component in the products intended for vaginal and/or ocular delivery [13,14,15,16,17,18,19]. Taurine is found in human uterine fluid, and taurine eye drops are commercially available, indicating its suitability for vaginal and ocular delivery [20,21,22]. Hence, we decided to evaluate the ability of maleic acid, fumaric acid, tartaric acid, citric acid, and taurine to form BX795-organic acid salt/cocrystals/co-amorphous systems. BX795 hydrochloride was also synthesized for comparison. We used a solvent-evaporation method to develop BX795-organic acid coevaporates followed by PXRD characterization to determine their solid-state characteristics. The PXRD of BX795-taurine coevaporate, and BX795-maleic acid coevaporate confirmed their crystalline nature, BX795-fumaric acid coevaporate showed partially crystalline behavior, and BX795-tartaric acid coevaporate and BX795-citric acid coevaporate showed the formation of a co-amorphous system. It is well known that the difference in the pK_a_ values of an ionizable developmental compound and counterion considerably influence the formation of salt/cocrystal/co-amorphous systems. Due to the lack of reported pKa values for BX795, we used MarvinSketch 21.2 to determine the theoretical pK_a_ values of BX795. The theoretical basic pK_a_ of BX795 predicted by MarvinSketch 21.2 was 3.68. The pK_a_ values of hydrochloride, taurine, maleic acid, fumaric acid, tartaric acid, and citric acid were obtained from the literature [23,24] to determine the difference in the pKa values (Δp*K*_a_) of BX795 and various counterions used in the study (Table 4). It is well regarded that Δp*K*_a_ value of >2 between drug and the counterion is likely to result in the formation of salt [25]. Hence, considering the difference in pK_a_ values between hydrochloride/taurine and BX795 (Table 4), BX795 hydrochloride and BX795 taurine can be regarded as salt. Typically, Δp*K*_a_ of <1 indicates the formation of neutral cocrystal involving hydrogen bond interactions between the drug and the conformer, and Δp*K*_a_ in the range of 1 to 3 represents intermediate protonation states [26]. The Δp*K*_a_ of 1.74 obtained for BX795-maleic acid coevaporate is greater than the generally accepted Δp*K*_a_ value necessary to form cocrystals, yet the XRPD of BX795-maleic acid shows adequate crystallinity. To further confirm the absence of ionization of maleic acid, we compared the FT-IR absorbance spectra of BX795, BX795-maleic acid coevaporate, maleic acid, and disodium maleate (Figure S1). BX795-maleic acid coevaporate showed the presence of an absorption band at 1709 cm^−1^ which is a characteristic of maleic acid and not disodium maleate indicating the absence of ionization of maleic acid in BX795-maleic acid coevaporate. It may be possible that BX795-maleic acid coevaporate is a partially ionized system maleate monoion that interacts with BX795. Due to the unavailability of maleate monoionic salt, we could not confirm the presence of partially ionized system. On the other hand, the Δp*K*_a_ of 0.64 obtained for the BX795-fumaric acid system indicated the possibility of cocrystal formation. However, the XRPD of BX795-fumaric acid coevaporate indicated partial amorphization. It is difficult to conclude whether BX795-fumaric acid coevaporate is a cocrystal or co-amorphous system. It could also be a heterogenous system consisting of BX795-fumaric acid co-amorphous mixture and fumaric acid crystal. The pK_a_ difference between BX795 and tartaric acid or citric acid is <1. Hence, the formation of salt was not anticipated in the case of BX795-tartaric acid/citric acid coevaporate. The PXRD of BX795-tartaric acid/citric acid coevaporate showed complete amorphization indicating the formation of co-amorphous systems. Furthermore, analysis of FT-IR spectra of BX795, BX795-tartaric acid/citric acid coevaporate, tartaric acid, disodium tartrate, citric acid, and trisodium citrate confirmed the lack of ionization of tartaric acid/citric acid in their respective co-amorphous systems with BX795 (Figure S1). The development of co-amorphous systems is an emerging amorphization strategy to improve the physicochemical and biopharmaceutical properties of the drug. The co-amorphous systems involve combining drug and low molecular weight organic excipient (the co-former) in an equimolar ratio using suitable methods to yield a homogenous amorphous single phase [27,28,29]. It should be noted that several studies have established the ability of citric acid and tartaric acid to form co-amorphous systems with weakly basic drugs [30,31,32,33], which corroborates our results on the formation of BX795-tartaric acid/citric acid co-amorphous systems. Furthermore, our PXRD analyses showed that BX795-tartaric acid/citric acid co-amorphous mixtures were stable for at least 1 month at room temperature (Figure S18). Our future studies would focus on the long-term physical and chemical stability of various BX795-organic acid salts/cocrystals/co-amorphous systems.

Organic counterions can improve various physicochemical properties including the thermal stability of ionizable developmental drugs. Our TGA studies showed that BX795 and BX795 hydrochloride underwent a 2% weight loss (T_2%_) at 36.39 and 55.32 °C, respectively (Table 3). Interestingly, the BX795-citric acid co-amorphous system showed the highest stability (T_2%_: 137.71 °C) followed by BX795 maleic acid (T_2%_: 116.37 °C), BX795 taurine (T_2%_: 91.36 °C), BX795 fumarate (T_2%_: 84.09 °C), and BX795 tartrate (T_2%_: 80.19 °C). This indicated the ability of counterions/coformers to improve the thermal stability of investigational drugs such as BX795.

Previous studies have also shown that different salt forms of the same drug can have dramatically different in vitro and in vivo performance [34,35,36]. Hence, it was necessary to measure the impact of the association of organic acids on the in vitro cytocompatibility and in vitro antiviral activity of BX795. Our initial in vitro cytocompatibility and in vitro antiviral activity screening involved evaluation of BX795 and BX795-organic acid salts/cocrystals/co-amorphous systems (all at a concentration equivalent to 10 µM BX795) to assess the impact of counterion/coformer on the biological activity of BX795. While all BX795-organic acid salts/cocrystals/co-amorphous systems showed similar or higher antiviral activity compared to BX795, only the BX795-citric acid co-amorphous system, BX795-tartaric acid co-amorphous system, and BX795 fumarate system showed at least similar or better in vitro cytocompatibility compared to BX795 in HCE and HeLa cells. Hence, we carried out dose-dependent in vitro cytocompatibility and in vitro antiviral activity studies on BX795, BX795-citric acid co-amorphous system, BX795-tartaric acid co-amorphous system, and BX795 fumarate system. Among various BX795 systems tested, the BX795-citric acid co-amorphous system showed higher in vitro cytocompatibility especially with HeLa cells (CC_50_: ~50 µM) compared to BX795 (CC_50_: ~25 µM) without compromising the in vitro antiviral activity against HSV-1 and HSV-2.

Citric acid or citric-acid-based buffers are used in ophthalmic preparations such as HyloGel^®^, HydraSense^®^, and citric acid is a component of vaginal gel Phexxi^®^, indicating its suitability for vaginal and ocular preparations. Hence, the BX795-citric acid co-amorphous system warrants further long-term characterization to enable the development of clinically viable topical vaginal and ocular formulations of BX795 for the improved treatment of genital and ocular herpes.

## 5. Conclusions

The organic acids with prior history of use for vaginal and ocular delivery, depending upon their pK_a_, show significant but differential interactions with weakly basic BX795 leading to the formation of salt/cocrystals/heterogenous mixtures/co-amorphous systems with improved thermal stability. In particular, the BX795-citric acid co-amorphous system, due to its greater in vitro cytocompatibility and unaltered in vitro antiviral activity has the potential for further pharmaceutical development.

## Figures and Tables

**Table 1:** Major FTIR frequencies (cm^−1^) showing shifts observed in BX795-organic acid coevaporates.

	O–H (Acid)	N–H Stretch	C=O (Acid)	N–H Bend	C–N (Amine)	S=O
**BX795**	-	3231	-	1530	1286	-
**Tartaric acid**	3401	-	1710	-	-	-
**BX795-tartaric acid**	2500–3300	3241	1713	1535	1296	-
**Maleic acid**	3320	-	1702	-	-	-
**BX795-maleic acid**	2500–3300	3240	1710	1544	1167	-
**Citric acid**	3493	-	1748	-	-	-
**BX795-citric acid**	2500–3300	3330	1731	1558	1302	-
**Fumaric acid**	3082	-	1668	-	-	-
**BX795-fumaric acid**	2500–3300	3237	1708	1538	1284	-
**Taurine**	-	-	-	-	-	1169
**BX795-taurate**	-	3207	-	1555	1303	1173
**BX795-HCl**	-	3222	-	1550	1297	-

**Table 2:** Proton shifts observed in the NMR spectra of BX795-organic acid coevaporates.

BX795	–COOH (ppm)	–NH (*δ*_H_: 8.59 ppm)	–CH (Pyrimidine) (*δ*_H_/*δ*_C_: 8.22/149.8)	–CH (Pyrrolidine) (*δ*_H_/*δ*_C_: 3.54/40.20)
**Citric acid**	12.40	-	-	-
**BX795-citric acid**	Disappeared	8.61	Disappeared	3.54/40.22
**Fumaric acid**	13.14	-	-	-
**BX795-fumaric acid**	Disappeared	8.59	Disappeared	3.54/40.20
**Maleic acid**	11.60	-	-	-
**BX795-maleic acid**	Disappeared	8.59	Disappeared	3.54/40.16
**Tartaric acid**	12.70	-	-	-
**BX795-tartaric acid**	Disappeared	8.61	Disappeared	3.54/40.23
**Taurine**	-	-	-	-
**BX795-taurate**	-	8.59	Disappeared	3.52/40.16
**BX795-HCl**	-	8.65	8.28/148.4	3.56/40.37

**Table 3:** Thermal stability of BX795 and BX795-organic acid systems (BX-HCl: BX795-dihydrochloride; BX-M: BX795-maleic acid; BX-Ci: BX795-citric acid; BX-Tau: BX795-taurate; BX-F: BX795-fumaric acid; BX-T: BX795-tartaric acid).

Mass Loss (%)	Temperature Corresponding to the % Mass Loss (°C)						
	BX795	BX-HCl	BX-M	BX-Ci	BX-Tau	BX-F	BX-T
−2	36.49	55.32	116.37	137.71	91.36	84.09	80.19
−5	195.9	173.31	134.97	161.06	227.44	174.05	171.27
−10	241.99	241.28	165.82	172.85	260.87	189.51	199.83
−20	-	292.59	264.87	201.61	-	245.16	262.49

**Table 4:** The pK_a_ values of the most acidic site in organic acids used in this investigation and the difference between the pK_a_ value of organic acid and the most basic site in BX795.

Organic Acid	pK_a,acid_	Δp*K*_a_ = p*K*_a,BX795_ − p*K*_a,acid_
**HCl**	−5.9	9.58
**Tartaric acid**	2.9	0.78
**Citric acid**	2.79	0.89
**Fumaric acid**	3.03	0.65
**Maleic acid**	1.94	1.74
**Taurine**	1.5	2.18

BX795 pK_a_: 3.68; MarvinSketch 22.1 was used to calculate the pKa of the most basic site of BX795.

## Data Availability

The main data supporting the findings of this study are available within the paper and its Supplementary Information. The associated raw data are available from the corresponding author on reasonable request.

## Supplementary Materials

The following are available online at https://www.mdpi.com/article/10.3390/pharmaceutics13111920/s1, Figure S1: FTIR spectra of BX795, pure organic acids, salts of organic acids, and BX795-organic acid coevaporates. Figure S2: The Raman spectra of BX795, pure organic acids, and BX795-organic acid coevaporates. Figures S3–S16: ^1^H and HSQC NMR spectra of BX795 and BX795-organic acid coevaporates. Figure S17: Cytotoxicity and in vitro biological activity of organic acids. Figure S18: PXRD profile of BX795 and BX795-organic acid coevaporates after 1-month storage.

## References

[1] Koganti R., Yadavalli T. (2019). Current and Emerging Therapies for Ocular Herpes Simplex Virus Type-1 Infections. Microorganisms.

[2] Lobo A.M., Agelidis A.M., Shukla D. (2019). Pathogenesis of herpes simplex keratitis: The host cell response and ocular surface sequelae to infection and inflammation. Ocul. Surf..

[3] Jaishankar D., Shukla D. (2016). Genital Herpes: Insights into Sexually Transmitted Infectious Disease. Microb. Cell.

[4] Jaishankar D., Yakoub A.M., Yadavalli T. (2018). An off-target effect of BX795 blocks herpes simplex virus type 1 infection of the eye. Sci. Transl. Med..

[5] Yadavalli T., Suryawanshi R., Ali M., Iqbal A., Koganti R., Ames J., Aakalu V.K., Shukla D. (2020). Prior inhibition of AKT phosphorylation by BX795 can define a safer strategy to prevent herpes simplex virus-1 infection of the eye. Ocul. Surf..

[6] Iqbal A., Suryawanshi R., Yadavalli T., Volety I., Shukla D. (2020). BX795 demonstrates potent antiviral benefits against herpes simplex Virus-1 infection of human cell lines. Antivir. Res..

[7] Hopkins J., Yadavalli T., Suryawanshi R., Qatanani F., Volety I., Koganti R., Iqbal A., Shukla D. (2020). In Vitro and In Vivo Activity, Tolerability, and Mechanism of Action of BX795 as an Antiviral against Herpes Simplex Virus 2 Genital Infection. Antimicrob. Agents Chemother..

[8] Saal C., Becker A. (2013). Pharmaceutical salts: A summary on doses of salt formers from the Orange Book. Eur. J. Pharm. Sci..

[9] Elder D.P., Holm R., Diego H.L. (2013). Use of pharmaceutical salts and cocrystals to address the issue of poor solubility. Int. J. Pharm..

[10] Berry D.J., Steed J.W. (2017). Pharmaceutical cocrystals, salts and multicomponent systems; intermolecular interactions and property based design. Adv. Drug Deliv. Rev..

[11] Bharate S.S. (2021). Recent developments in pharmaceutical salts: FDA approvals from 2015 to 2019. Drug Discov. Today.

[12] Yadavalli T., Ames J. (2019). Drug-encapsulated carbon (DECON): A novel platform for enhanced drug delivery. Sci. Adv..

[13] Aguirre S.A., Collette W., Gukasyan H.J., Huang W. (2012). An assessment of the ocular safety of excipient maleic acid following intravitreal injection in rabbits. Toxicol. Pathol..

[14] Cazorla-Luna R., Martín-Illana A., Notario-Pérez F., Bedoya L.M. (2020). Vaginal Polyelectrolyte Layer-by-Layer Films Based on Chitosan Derivatives and Eudragit® S100 for pH Responsive Release of Tenofovir. Mar. Drugs.

[15] Oh D.J., Chen J.L., Vajaranant T.S., Dikopf M.S. (2019). Brimonidine tartrate for the treatment of glaucoma. Expert Opin. Pharmacother..

[16] https://phexxi.com/?gclid=EAIaIQobChMI-f2Q94-K8QIVSOvjBx2ulAeZEAAYASAAEgJjBvD_BwE&gclsrc=aw.ds.

[17] Kar D. How Hyaluronic Acid Eyedrops Fit into Dry Eye Treatment. https://eyesoneyecare.com/resources/how-hyaluronic-acid-eyedrops-fit-dry-eye-treatment/.

[18] Pfister R.R., Haddox J.L., Yuille-Barr D. (1991). The combined effect of citrate/ascorbate treatment in alkali-injured rabbit eyes. Cornea.

[19] Schuerer N., Stein E., Inic-Kanada A., Pucher M., Hohenadl C., Bintner N., Ghasemian E., Montanaro J., Barisani-Asenbauer T. (2017). Implications for Ophthalmic Formulations: Ocular Buffers Show Varied Cytotoxic Impact on Human Corneal-Limbal and Human Conjunctival Epithelial Cells. Cornea.

[20] Devreker F., Van den Bergh M., Biramane J., Winston R.L., Englert Y., Hardy K. (1999). Effects of taurine on human embryo development in vitro. Hum. Reprod..

[21] Nor Arfuzir N.N., Agarwal R., Iezhitsa I., Agarwal P., Sidek S., Ismail N.M. (2018). Taurine protects against retinal and optic nerve damage induced by endothelin-1 in rats via antioxidant effects. Neural. Regen. Res..

[22] Advacare Pharma Taurine Eye Drops (TarinCare™). https://www.advacarepharma.com/en/pharmaceuticals/taurine-eye-drops.

[23] Fung M.H., DeVault M., Kuwata K.T., Suryanarayanan R. (2018). Drug-Excipient Interactions: Effect on Molecular Mobility and Physical Stability of Ketoconazole-Organic Acid Coamorphous Systems. Mol. Pharm..

[24] Black S.N., Collier E.A., Davey R.J., Roberts R.J. (2007). Structure, solubility, screening, and synthesis of molecular salts. J. Pharm. Sci..

[25] Karagianni A., Kachrimanis K., Nikolakakis I. (2018). Co-Amorphous Solid Dispersions for Solubility and Absorption Improvement of Drugs: Composition, Preparation, Characterization and Formulations for Oral Delivery. Pharmaceutics.

[26] Nie H., Byrn S.R., Zhou Q.T. (2017). Stability of pharmaceutical salts in solid oral dosage forms. Drug Dev. Ind. Pharm..

[27] Joshi M., Roy Choudhury A. (2018). Salts of Amoxapine with Improved Solubility for Enhanced Pharmaceutical Applicability. ACS Omega.

[28] Han J., Wei Y., Lu Y., Wang R., Zhang J., Gao Y., Qian S. (2020). Co-amorphous systems for the delivery of poorly water-soluble drugs: Recent advances and an update. Expert Opin. Drug Deliv..

[29] Liu J., Grohganz H. (2021). Co-Amorphous Drug Formulations in Numbers: Recent Advances in Co-Amorphous Drug Formulations with Focus on Co-Formability, Molar Ratio, Preparation Methods, Physical Stability, In Vitro and In Vivo Performance, and New Formulation Strategies. Pharmaceutics.

[30] Hirakawa Y., Ueda H., Takata Y., Minamihata K., Wakabayashi R., Kamiya N., Goto M. (2021). Co-amorphous formation of piroxicam-citric acid to generate supersaturation and improve skin permeation. Eur. J. Pharm. Sci..

[31] Wu W., Ueda H., Löbmann K., Rades T., Grohganz H. (2018). Organic acids as co-formers for co-amorphous systems-Influence of variation in molar ratio on the physicochemical properties of the co-amorphous systems. Eur. J. Pharm. Biopharm..

[32] Ueda H., Wu W., Löbmann K., Grohganz H. (2018). Application of a Salt Coformer in a Co-Amorphous Drug System Dramatically Enhances the Glass Transition Temperature: A Case Study of the Ternary System Carbamazepine, Citric Acid, and l-Arginine. Mol. Pharm..

[33] An J.H., Lim C., Kiyonga A.N., Chung I.H., Lee I.K., Mo K., Park M., Youn W., Choi W.R., Suh Y.G. (2018). Co-Amorphous Screening for the Solubility Enhancement of Poorly Water-Soluble Mirabegron and Investigation of Their Intermolecular Interactions and Dissolution Behaviors. Pharmaceutics.

[34] Bannigan P., Durack E., Madden C., Lusi M., Hudson S.P. (2017). Role of Biorelevant Dissolution Media in the Selection of Optimal Salt Forms of Oral Drugs: Maximizing the Gastrointestinal Solubility and in Vitro Activity of the Antimicrobial Molecule, Clofazimine. ACS Omega.

[35] Saeed H.K., Sutar Y., Patel P., Bhat R., Mallick S., Hatada A.E., Koomoa D.T., Lange I., Date A.A. (2021). Synthesis and Characterization of Lipophilic Salts of Metformin to Improve Its Repurposing for Cancer Therapy. ACS Omega.

[36] Patel A., Keir S.D., Brown M.B., Hider R., Jones S.A., Page C.P. (2016). Using Salt Counterions to Modify β(2)-Agonist Behavior in Vivo. Mol. Pharm..

