# Supplementary material for: BX795-Organic Acid Coevaporates: Evaluation of Solid-State Characteristics, In Vitro Cytocompatibility and In Vitro Activity against HSV-1 and HSV-2
Source: Pharmaceutics. 2021 Nov 12;13(11):1920. doi: 10.3390/pharmaceutics13111920 (PMC8623185; doi:10.3390/pharmaceutics13111920)
Supplement: Supplementary file 1 [file pharmaceutics-13-01920-s001.zip › pharmaceutics-1352871-supplementary.pdf]

pharmaceutics

MDPI

Supplementary Materials: BX795-organic Acid Coevaporates: Evaluation of Solid-State Characteristics, In Vitro Cytocompatibility and In Vitro Activity Against HSV-1 and HSV-2

Yogesh Sutar, Tejabhiram Yadavalli, Sagar Paul, Sudipta Mallick, Raghuram Koganti, Harsh Chauhan, Abhijit A. Date, Deepak Shukla

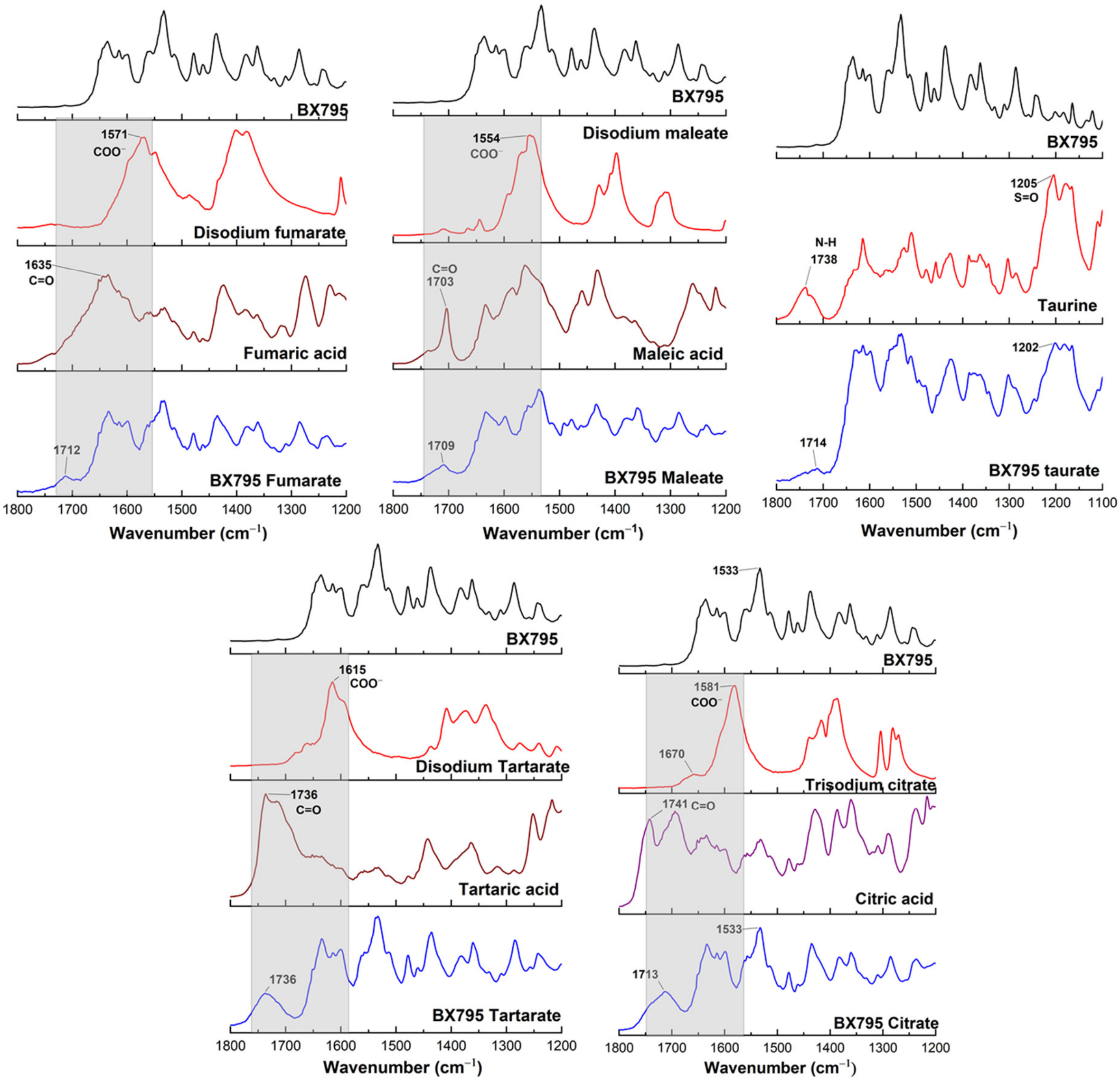

Figure S1. FTIR spectra of BX795, pure organic acids, salts of organic acids, and BX795-organic acid coevaporates.

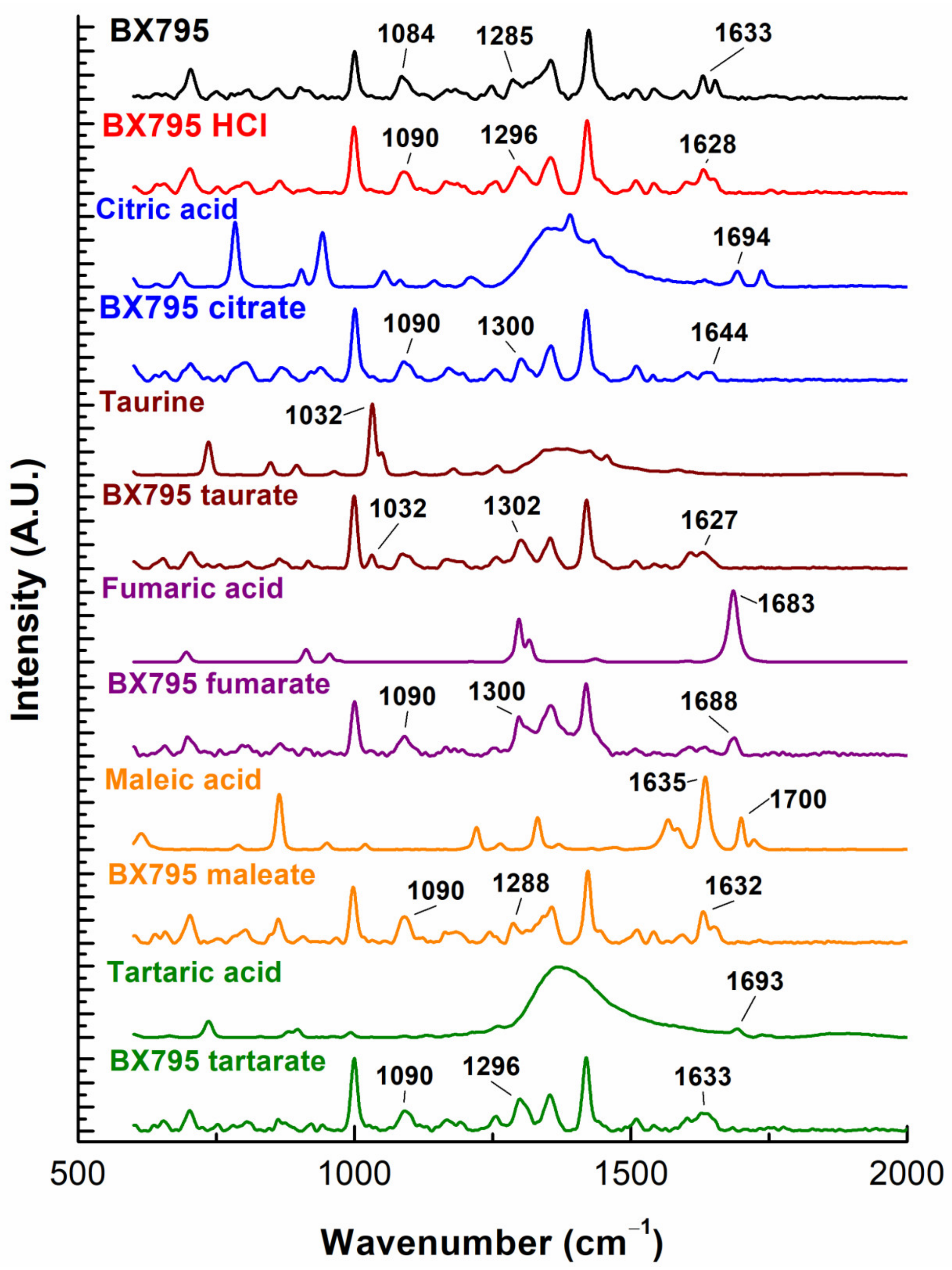

Figure S2. The Raman spectra of BX795, pure organic acids, and BX795-organic acid coevaporates. The Raman spectra confirm the interaction between BX795 and organic acids.

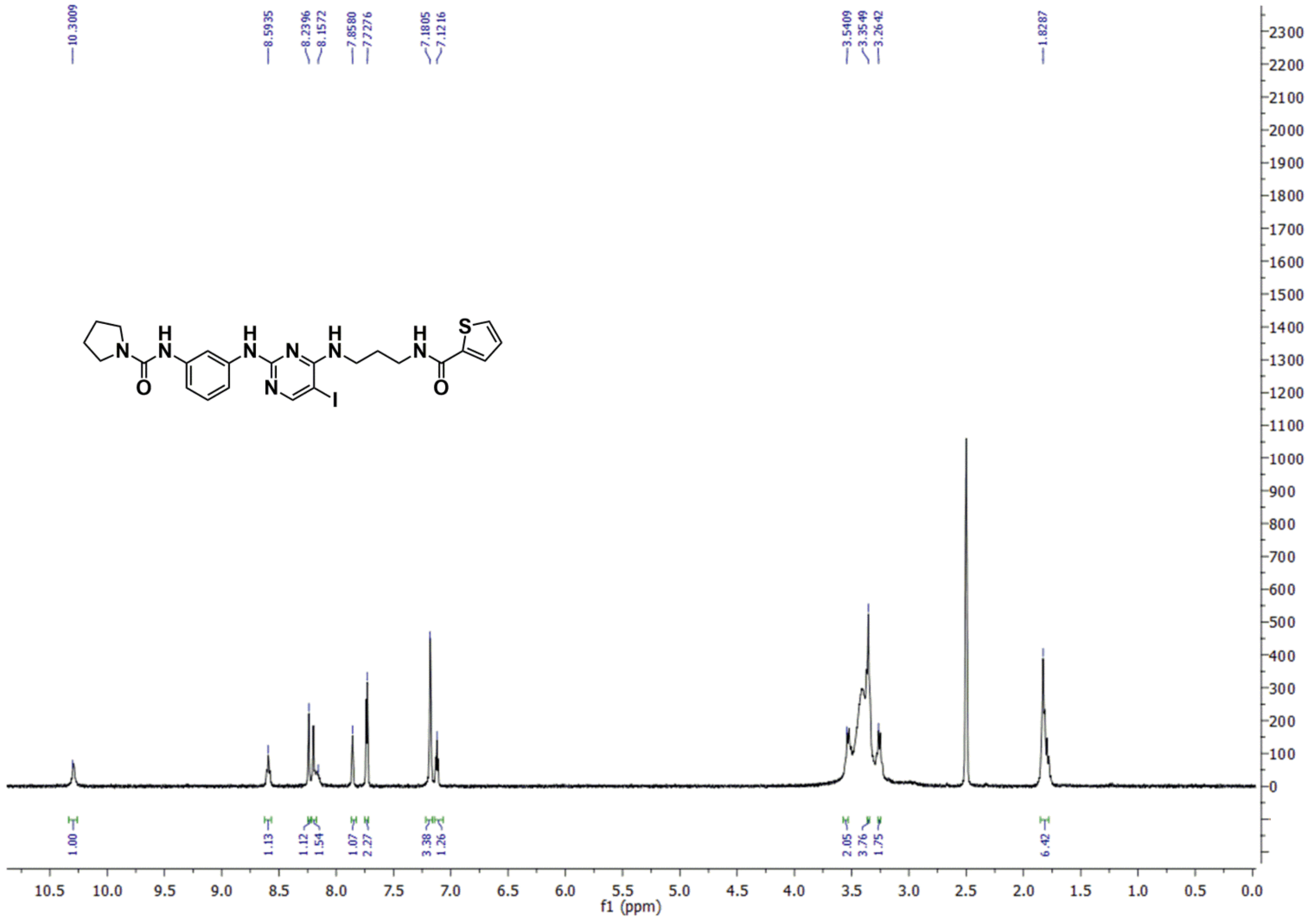

Figure S3. ^{1}H NMR Spectrum of BX795.

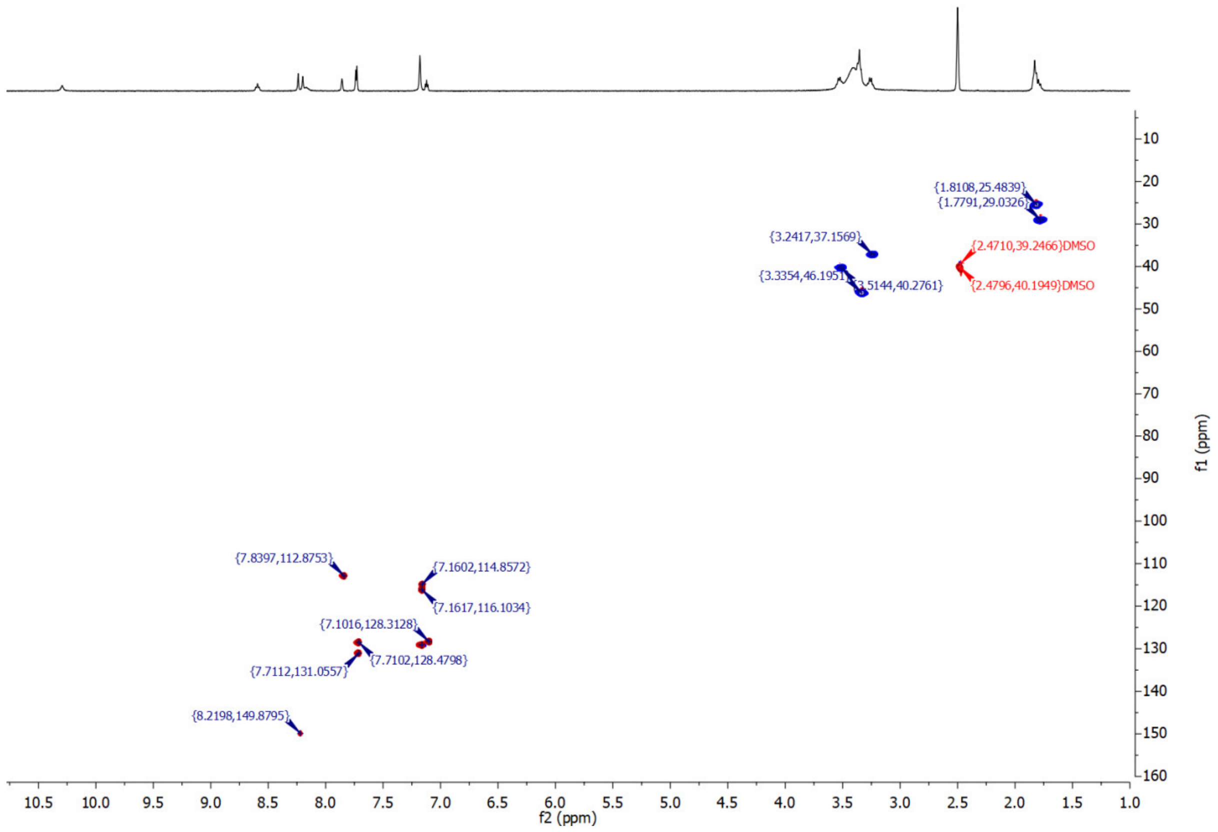

Figure S4. HSQC NMR Spectrum of BX795.

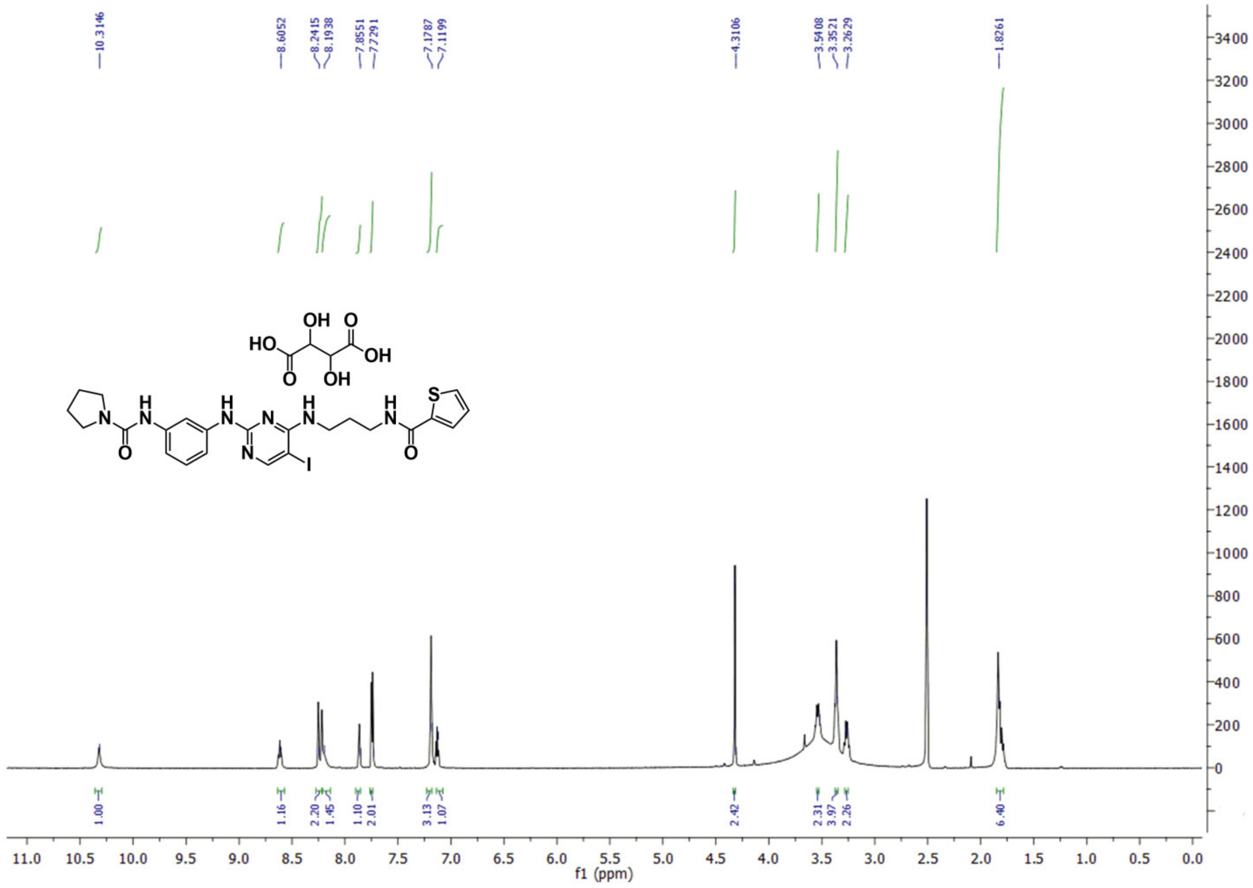

Figure S5. ^{1}H NMR Spectrum of BX795 tartarate.

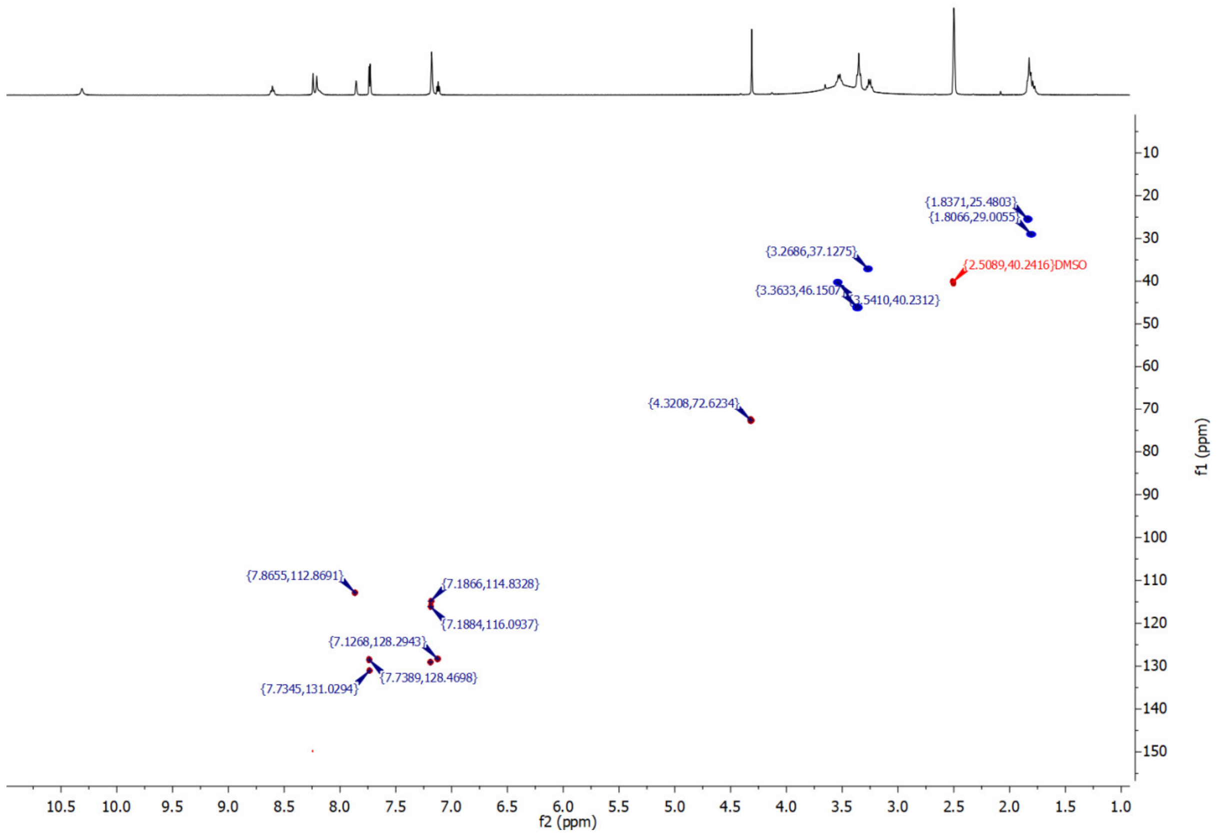

Figure S6. HSQC NMR Spectrum of BX795 tartarate.

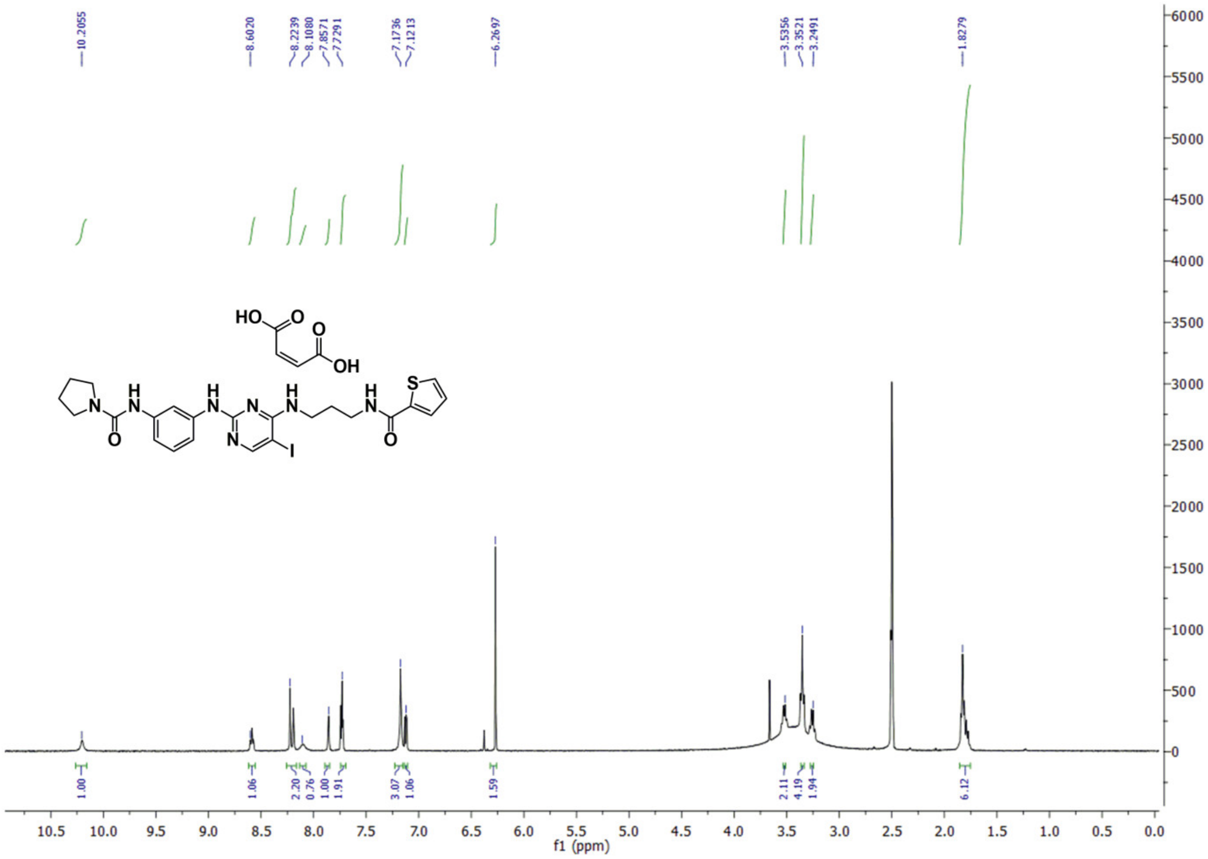

Figure S7. ¹H NMR Spectrum of BX795 maleate.

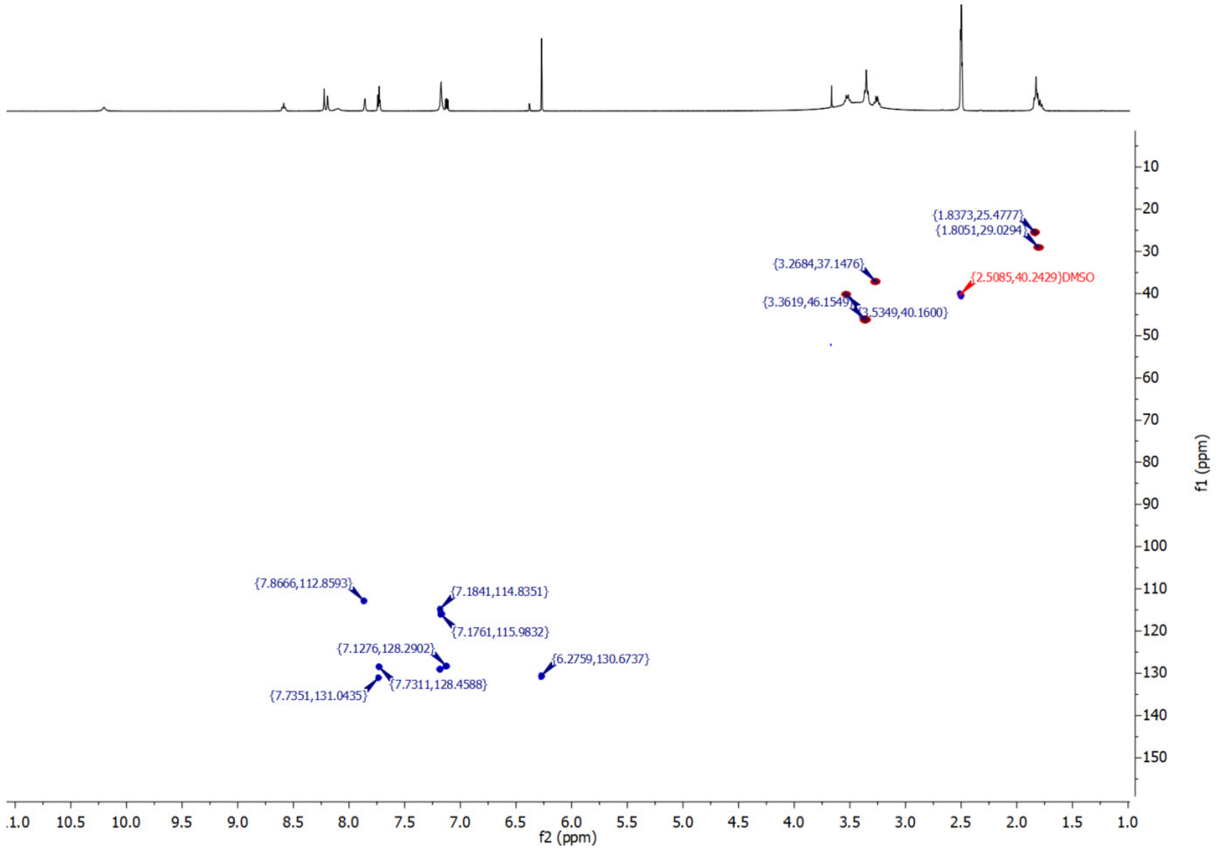

Figure S8. HSQC NMR Spectrum of BX795 maleate.

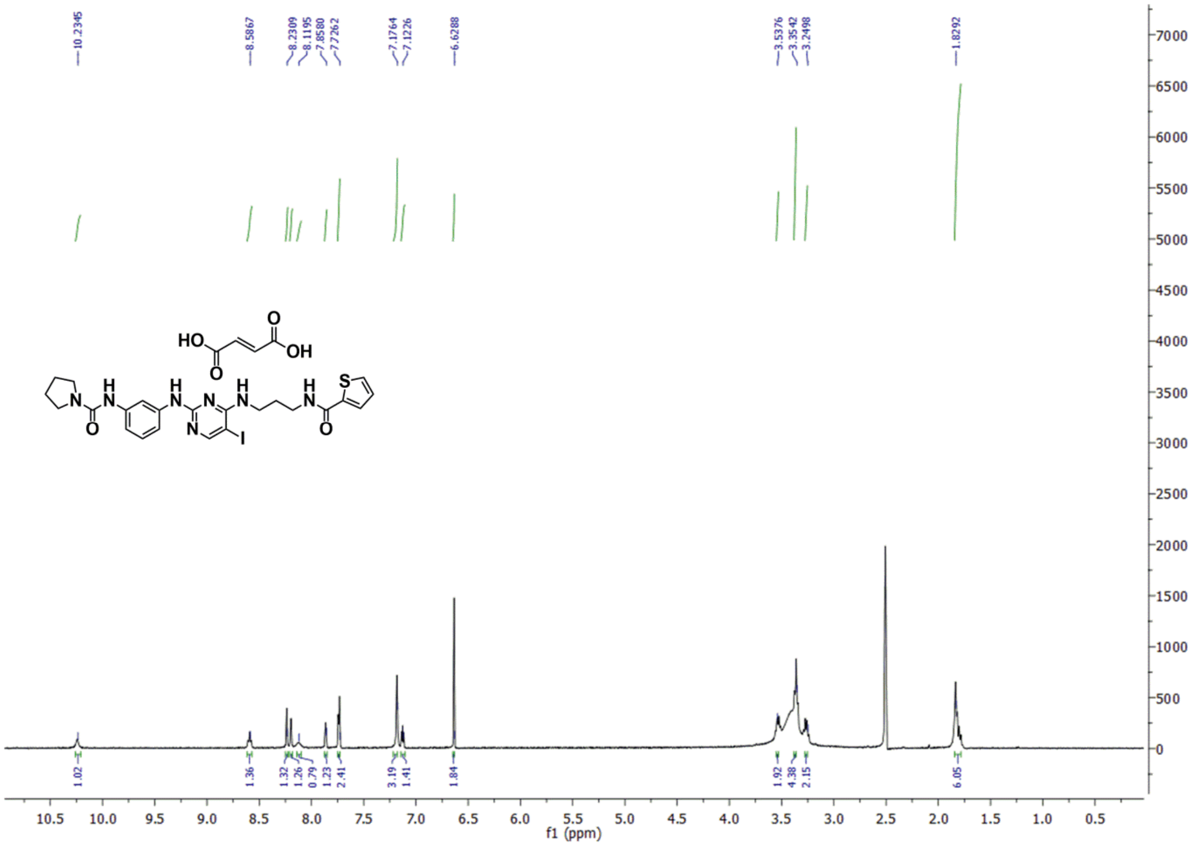

Figure S9. ^{1}H NMR Spectrum of BX795 fumarate.

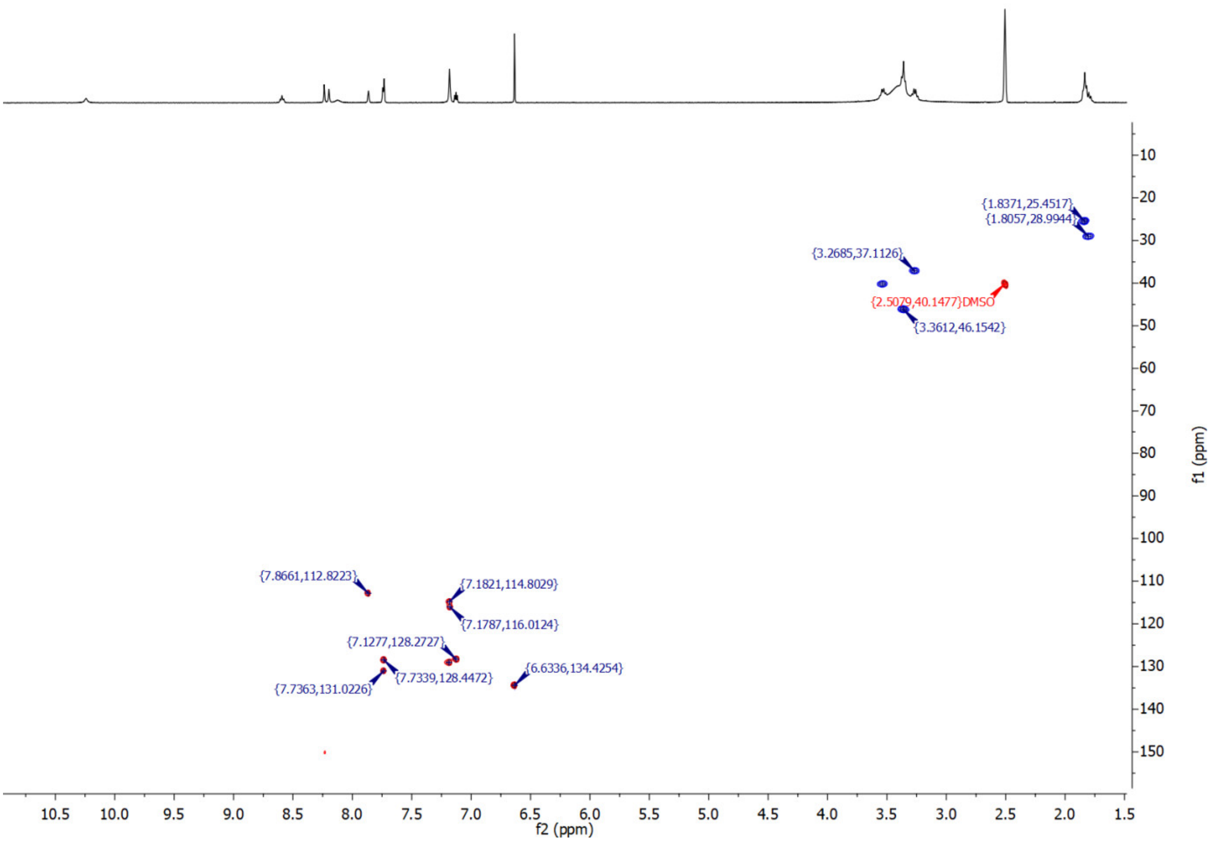

Figure S10. HSQC NMR Spectrum of BX795 fumarate.

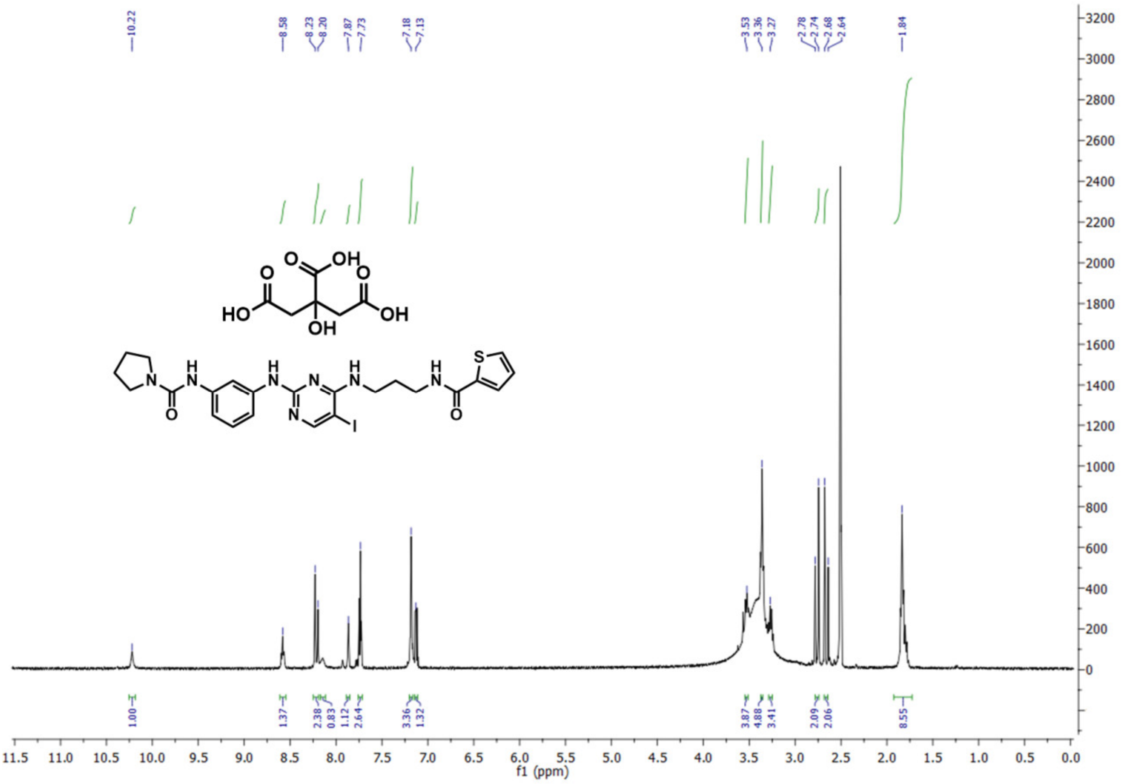

Figure S11. ^{1}H NMR Spectrum of BX795 citrate.

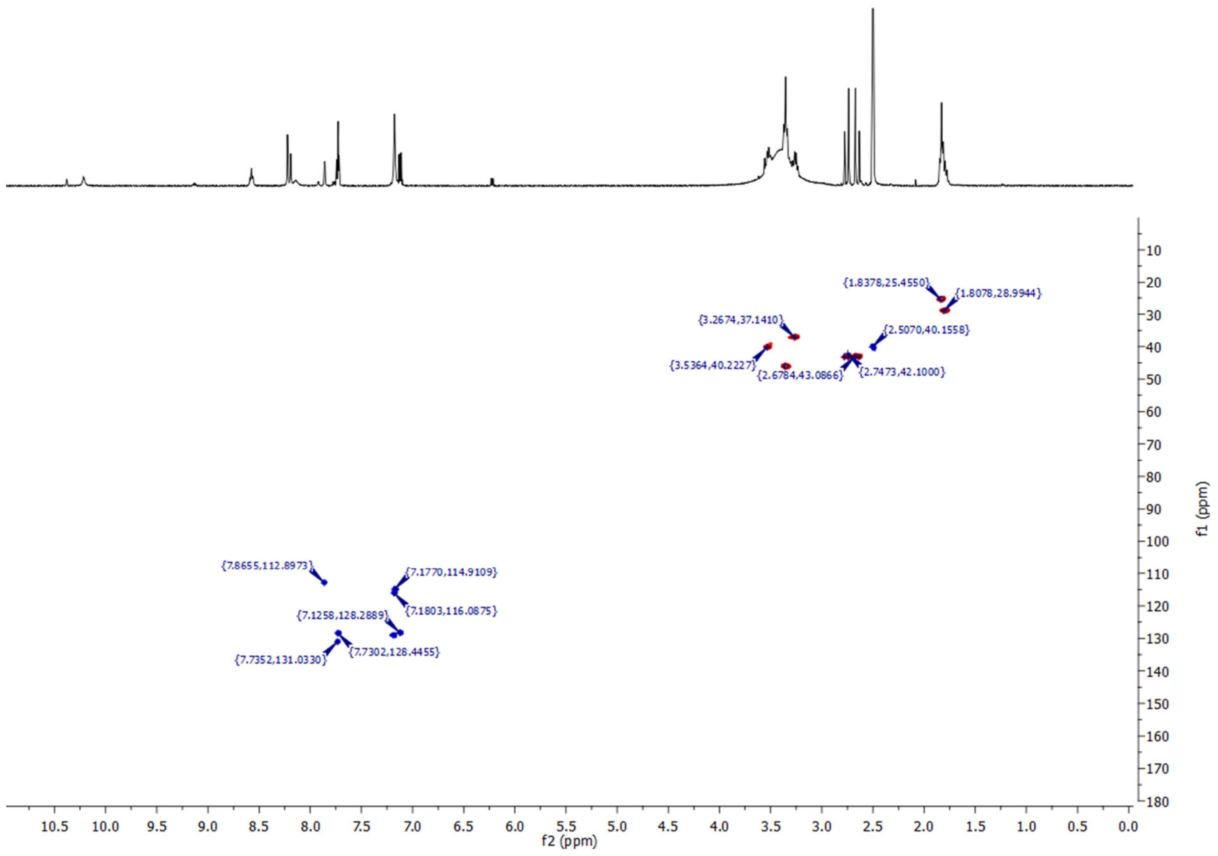

Figure S12. HSQC NMR Spectrum of BX795 citrate.

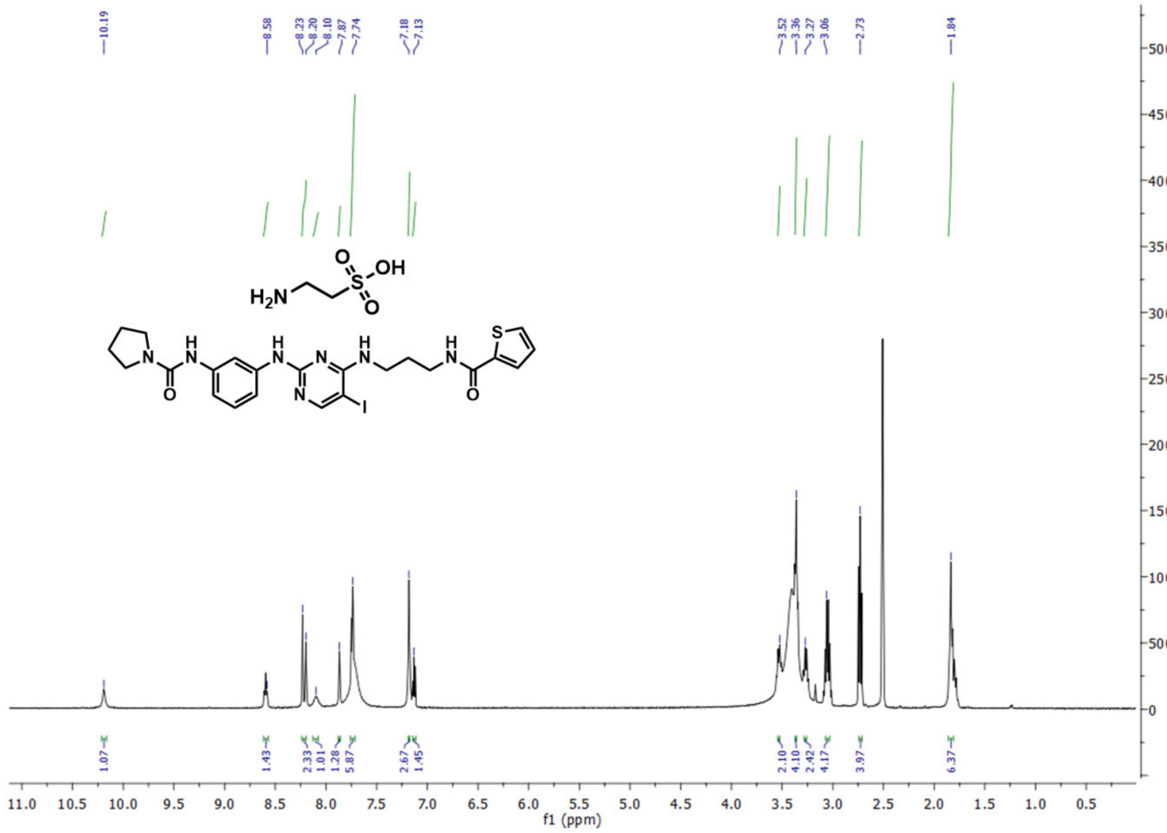

Figure S13. ^{1}H NMR Spectrum of BX795 taurate.

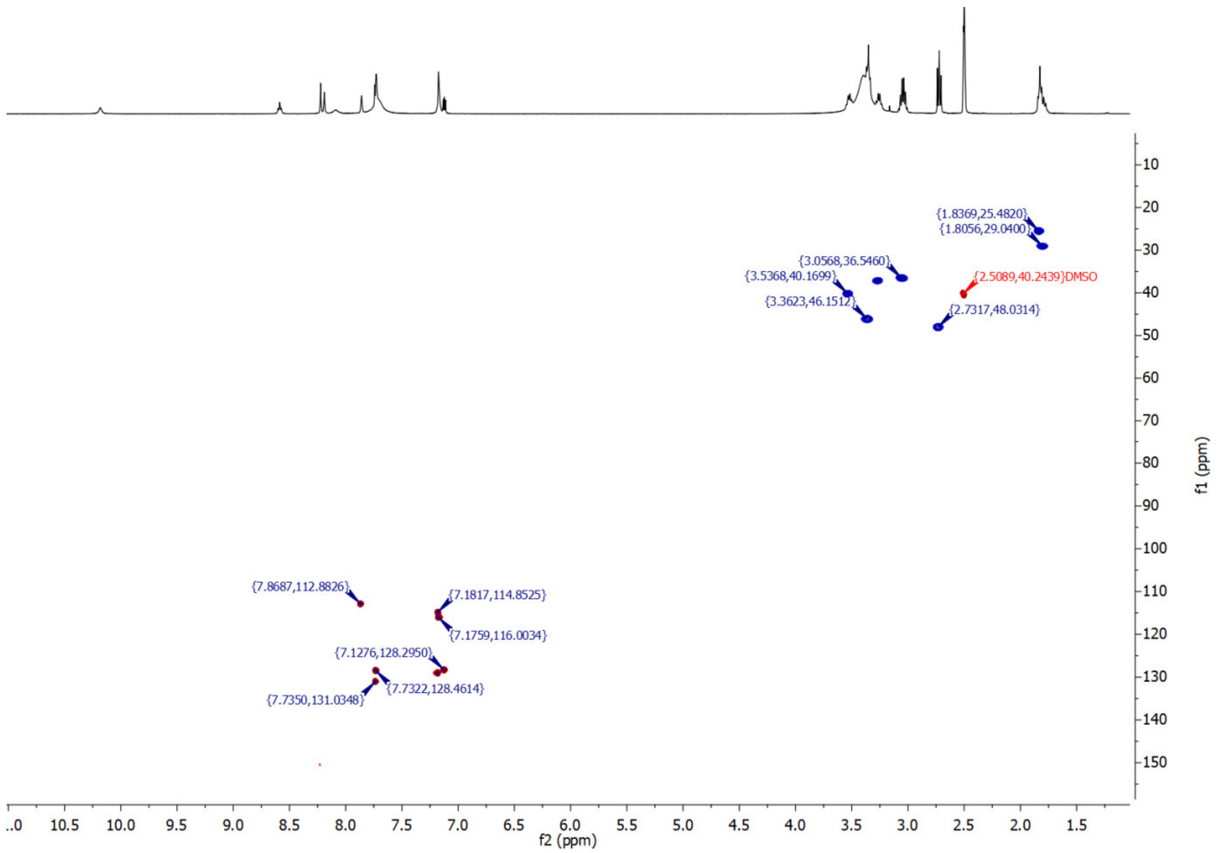

Figure S14. HSQC NMR Spectrum of BX795 taurate.

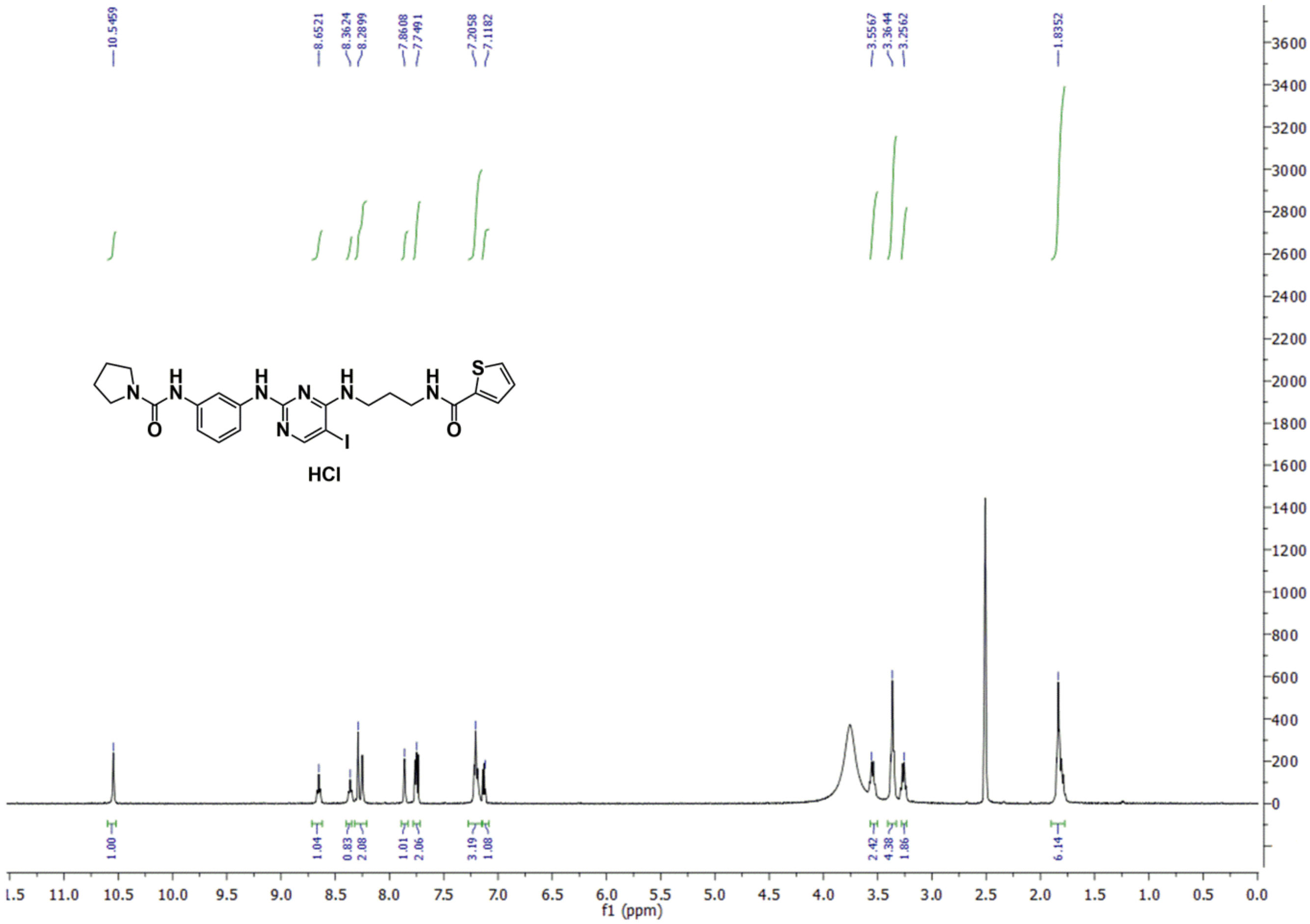

Figure S15. ^{1}H NMR Spectrum of BX795 hydrochloride.

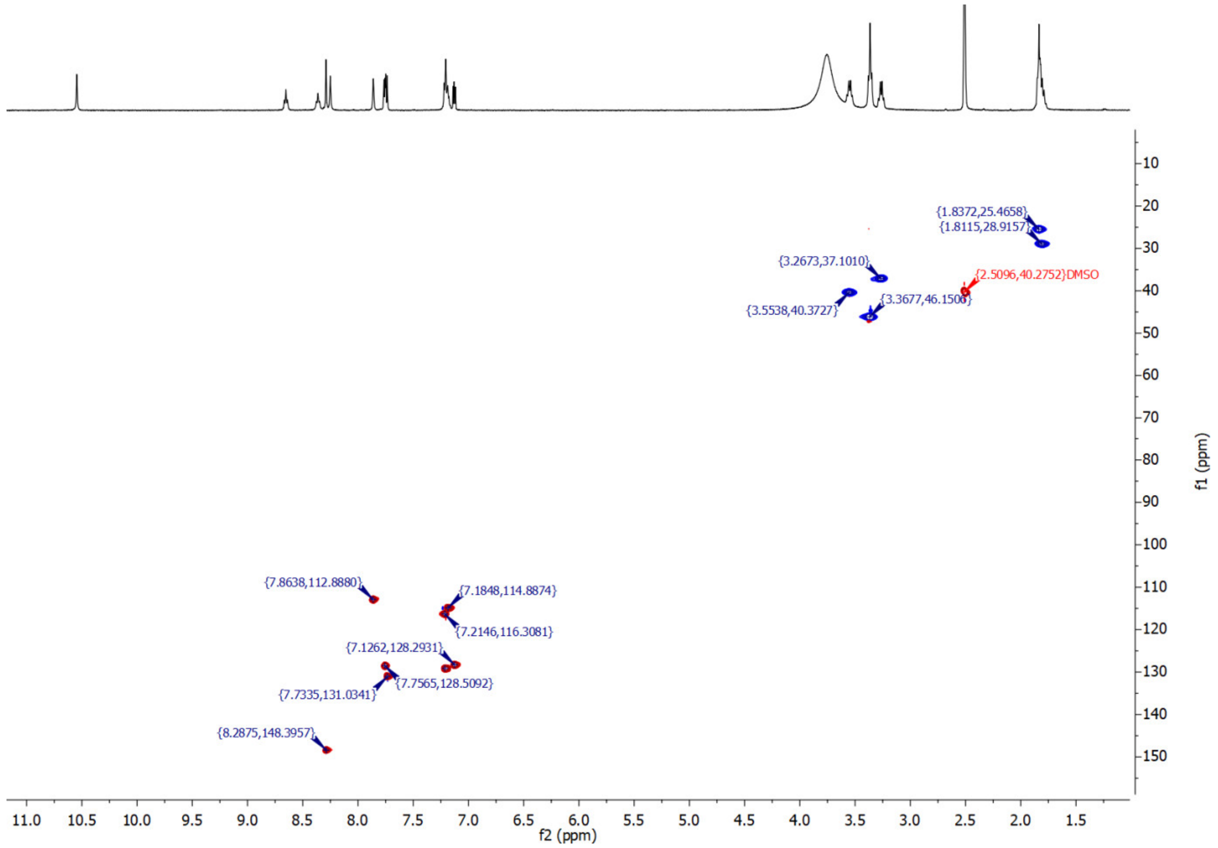

Figure S16. HSQC NMR Spectrum of BX795 hydrochloride.

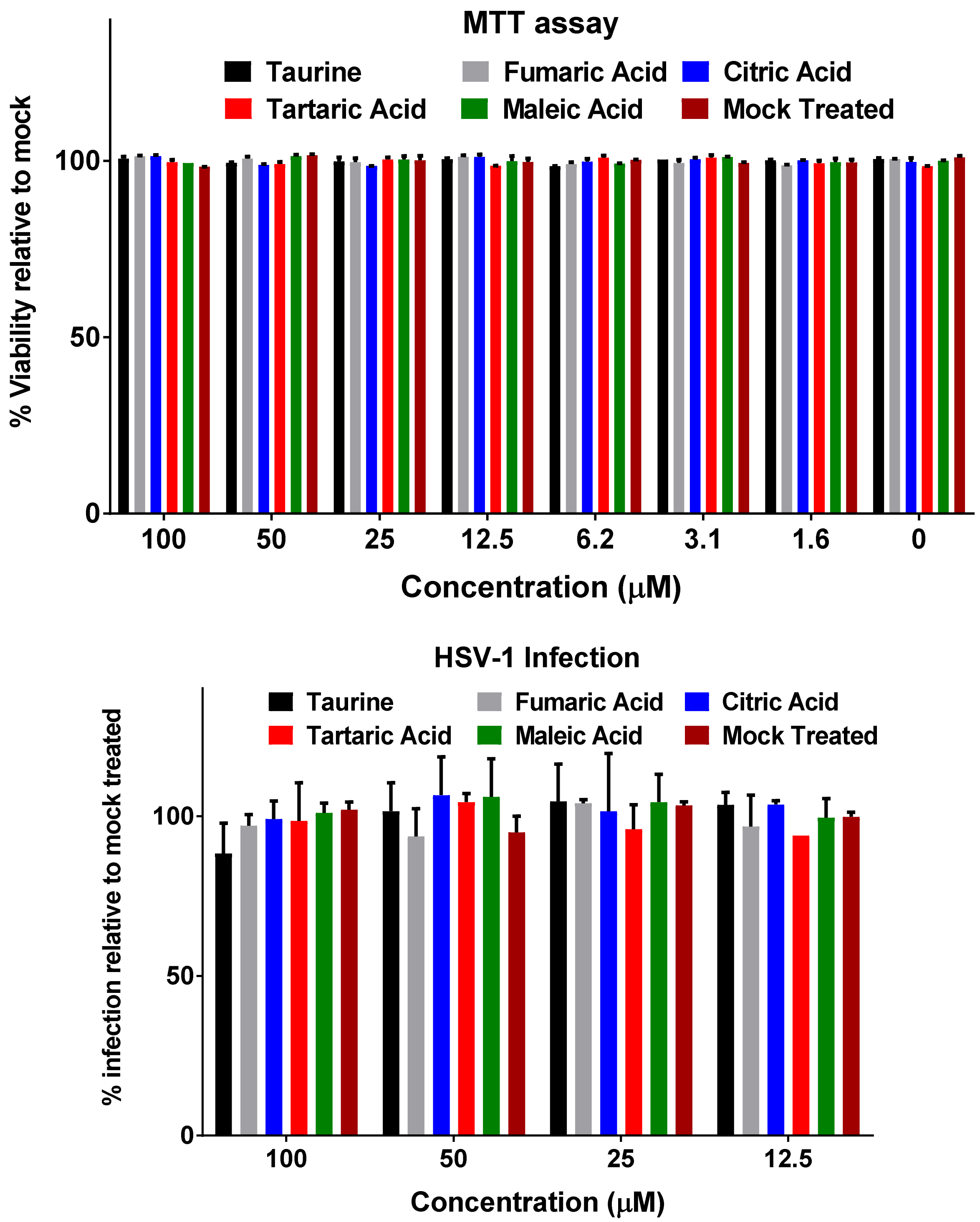

Figure S17. Organic acids used for the preparation of BX795-organic acid coevaporates did not show any cytotoxicity and antiviral activity at concentrations equivalent to those used for the in vitro biological activity testing of the BX795-organic acid coevaporates.

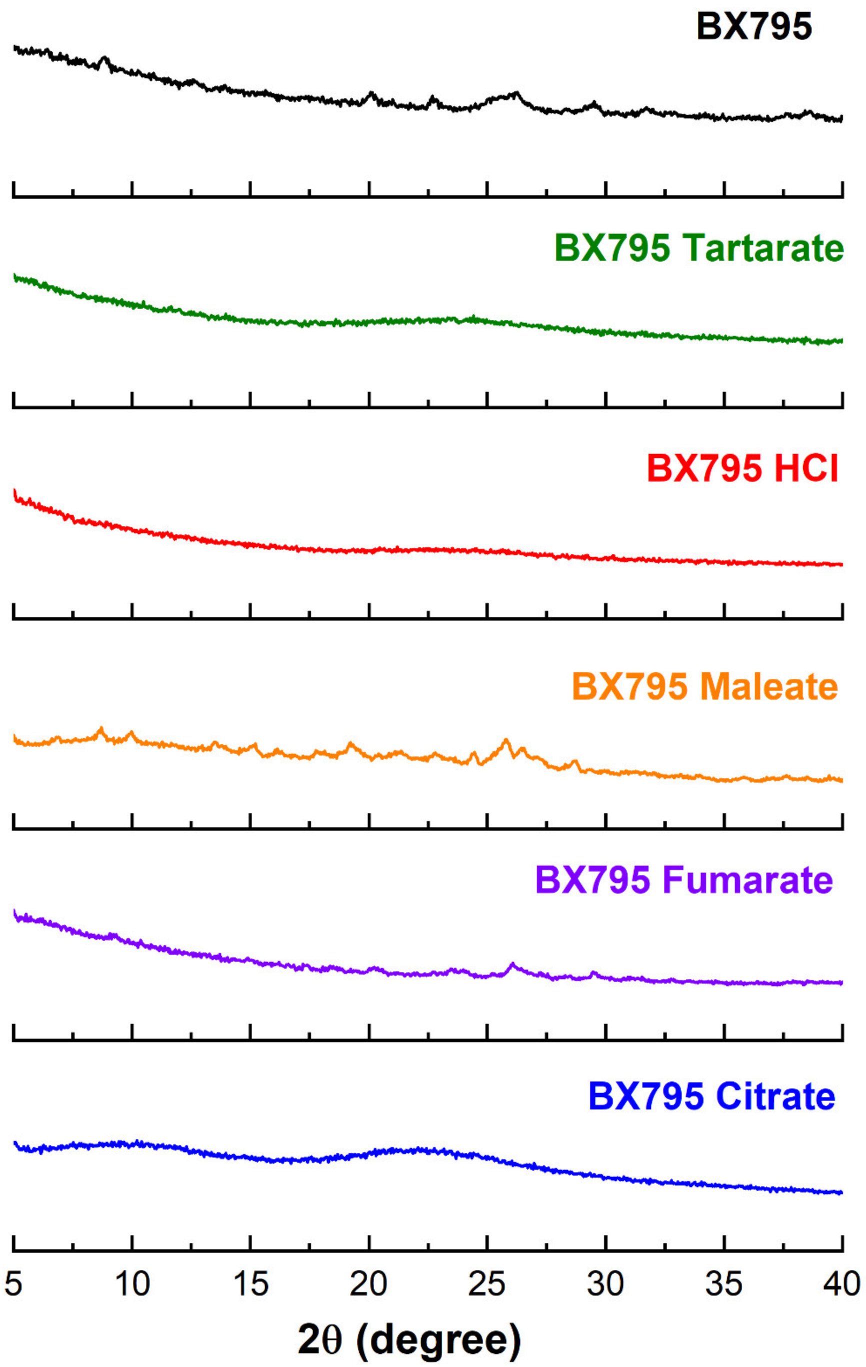

Figure S18. PXRD profile of BX795 and BX795-organic acid coevaporates after 1-month of storage at room temperature.
